# REBOARREST, resuscitative endovascular balloon occlusion of the aorta in non-traumatic out-of-hospital cardiac arrest: a study protocol for a randomised, parallel group, clinical multicentre trial

**DOI:** 10.1186/s13063-021-05477-1

**Published:** 2021-07-31

**Authors:** Jostein Rødseth Brede, Arne Kristian Skulberg, Marius Rehn, Kjetil Thorsen, Pål Klepstad, Ida Tylleskär, Bjørn Farbu, Jostein Dale, Trond Nordseth, Rune Wiseth, Andreas Jørstad Krüger

**Affiliations:** 1 Department of Emergency Medicine and Pre-Hospital Services, St. Olav’s Hospital, Trondheim University Hospital, Prinsesse Kristinas Gate 3, 7030 Trondheim, Norway; 2 Norwegian Air Ambulance Foundation, Department of Research and Development, Oslo, Norway; 3 Department of Anesthesiology and Intensive Care Medicine, St. Olav’s Hospital, Trondheim University Hospital, Prinsesse Kristinas Gate 3, 7030 Trondheim, Norway; 4 Department of Circulation and Medical Imaging, Faculty of Medicine and Health Sciences, Norwegian University of Science and Technology (NTNU), Trondheim, Norway; 5 Division of Prehospital Services, Air Ambulance Department, Oslo University Hospital, Oslo, Norway; 6 Faculty of Health Sciences, University of Stavanger, Stavanger, Norway; 7 Clinic of Cardiology, St. Olav’s Hospital, Trondheim University Hospital, Prinsesse Kristinas Gate 3, 7030 Trondheim, Norway

**Keywords:** Advanced cardiopulmonary resuscitation (ACLS), Aortic occlusion, Cardiac arrest, Cardiopulmonary resuscitation (CPR), Resuscitative endovascular balloon occlusion of the aorta (REBOA), Return of spontaneous circulation (ROSC)

## Abstract

**Background:**

Survival after out-of-hospital cardiac arrest (OHCA) is poor and dependent on high-quality cardiopulmonary resuscitation. Resuscitative endovascular balloon occlusion of the aorta (REBOA) may be advantageous in non-traumatic OHCA due to the potential benefit of redistributing the cardiac output to organs proximal to the aortic occlusion. This theory is supported by data from both preclinical studies and human case reports.

**Methods:**

This multicentre trial will enrol 200 adult patients, who will be randomised in a 1:1 ratio to either a control group that receives advanced cardiovascular life support (ACLS) or an intervention group that receives ACLS and REBOA. The primary endpoint will be the proportion of patients who achieve return of spontaneous circulation with a duration of at least 20 min. The secondary objectives of this trial are to measure the proportion of patients surviving to 30 days with good neurological status, to describe the haemodynamic physiology of aortic occlusion during ACLS, and to document adverse events.

**Discussion:**

Results from this study will assess the efficacy and safety of REBOA as an adjunctive treatment for non-traumatic OHCA. This novel use of REBOA may contribute to improve treatment for this patient cohort.

**Trial registration:**

The trial is approved by the Regional Committee for Medical and Health Research Ethics in Norway (reference 152504) and is registered at ClinicalTrials.gov (reference NCT04596514) and as Universal Trial Number WHO: U1111-1253-0322.

**Supplementary Information:**

The online version contains supplementary material available at 10.1186/s13063-021-05477-1.

## Background

### Current knowledge and practice

Out-of-hospital cardiac arrest (OHCA) has a high mortality rate [1]. Deaths in patients who survive until admission to hospital are most often the result of anoxic brain damage [2]. Cardiopulmonary resuscitation (CPR) prolongs the time until irreversible hypoxic damage occurs by delivering partially oxygenated blood to the brain and other vital organs [3]. The main treatment used for OHCA is advanced cardiovascular life support (ACLS) as set out in the guidelines published by organisations such as the Norwegian Resuscitation Council [4] and the European Resuscitation Council (ERC) [5].

### Adjuncts to advanced cardiovascular life support

In addition to ACLS, interventions such as fibrinolysis and percutaneous coronary intervention are recommended as appropriate in cardiac arrest [5]. Other possible interventions in the setting of cardiac arrest include extracorporeal membrane oxygenation [6]. Although currently not recommended in the ERC guidelines, some centres have implemented an extracorporeal CPR protocol. The REBOARREST trial is designed to assess the ability of resuscitative endovascular balloon occlusion of the aorta (REBOA) to increase the rate of return of spontaneous circulation (ROSC) when used as an adjunct to ACLS.

### Study rationale

REBOA is a technique whereby blood flow through the aorta is occluded by inflation of an intra-aortic balloon. REBOA is approved for management of haemorrhagic shock and traumatic cardiac arrest. It has recently been proposed as an adjunctive treatment for patients with non-traumatic cardiac arrest [7, 8] due to the potential benefit of redistributing the cardiac output to organs proximal to the occlusion. A growing body of preclinical evidence supports the hypothesis that patients with non-traumatic cardiac arrest might benefit from REBOA during CPR [9–16]. These studies have demonstrated increases in coronary artery blood flow, perfusion pressure, and rates of ROSC when REBOA is used during cardiac arrest. Coronary perfusion pressure is associated with ROSC in humans [17]. The studies also demonstrate increases in carotid artery blood flow [12, 18], blood flow in the cerebral arteries [10, 11, 18–20], and cerebral perfusion pressure [10, 11, 18, 21]. Moreover, a few case reports have indicated that REBOA has an effect in humans with cardiac arrest [22–25]. Currently, there is only one report on the prospective clinical use of REBOA in the pre-hospital setting [26]. This study showed that pre-hospital REBOA procedure during resuscitation is feasible and did not negatively influence the quality of ACLS.

There are known risks associated with REBOA from the use in haemorrhage treatment, such as arterial injuries or organ ischemia. OHCA is a different context and in that critical setting, the potential benefit of REBOA may outweigh or balance these risks. We anticipate that REBOA will increase blood pressure during both the compression and decompression phase of CPR, which could potentially improve perfusion of the brain and heart. Brain tissue is highly sensitive to hypoxemia; therefore, increased systolic blood pressure and improved perfusion of the brain is likely to be beneficial. Hence, the potential clinical benefit in this trial would include increased rates of ROSC and survival until admission to hospital and possibly also an improved rate of 30-day survival with a favourable neurological outcome.

## Methods/design

### Organisation and conduct

The study protocol is drafted in accordance with the SPIRIT (Standard Protocol Items: Recommendations for Interventional Trial) guidelines [27] (see Additional file 1) and will be reported in accordance with the CONSORT (Consolidated Standards of Reporting Trials) guidelines [28]. The protocol has been assessed by the Norwegian Medicines Agency and found not to be covered by the European Union regulation 2017/745 concerning medical devices. The trial sponsor is the Clinic of Cardiology, St. Olav’s Hospital, Trondheim University Hospital, Norway. The study is coordinated by the Norwegian Air Ambulance base in Trondheim, in cooperation with KlinForsk, Clinical Research Unit, Central Norway.

### Design

The primary objective of this prospective, multicentre, randomised, parallel group, clinical trial is to assess the efficacy of REBOA as an adjunctive treatment to ACLS in patients with OHCA. The primary endpoint is the proportion of patients that achieve ROSC with a duration of at least 20 min. The secondary objectives are to measure the proportion of patients surviving to 30 day with good neurological status, to describe the haemodynamic physiology of aortic occlusion during ACLS, and to document any adverse events. All endpoints are summarised in Table 1. Further information on the rationale for selection of endpoints can be found in the detailed study protocol, which is available as supplemental material (see Additional file 2) and on the trial website (www.reboarrest.com).

**Table 1:** Trial endpoints

Primary endpoint	Proportions of patients that achieve return of spontaneous circulation with a duration of at least 20 min
**Secondary endpoints**	The proportion of patients surviving to 30 days with good neurological status, defined as a modified Rankin scale score of 0–3
	Difference in end-tidal CO_2_ measurements in the control group and the intervention group after aortic occlusion
	Change in blood pressures after aortic occlusion
	Left ventricular ejection fraction (LVEF) measured by echocardiography
**Exploratory endpoints**	All-cause mortality 1 year after randomisation
	Difference in organ function, using the Acute Kidney Injury Network (AKIN) classification, liver function blood tests, and others
	Incidence of all adverse events

The study will enrol 200 patients (100 in each group) over a period of 3 years. A training program will be in place for all teams and all pre-hospital responders working at the study site will receive training [29]. Planned study sites are pre-hospital service providers in Norway, Denmark, and Sweden. Other countries may be added during the trial period.

### Eligibility criteria

The study population will consist of adults with OHCA assumed to be non-traumatic in origin as determined by the on-scene physician. All patients with OHCA at a study centre will be screened for inclusion. Patients who are not included in the study will be treated according to the local ACLS guideline at the study site. The inclusion and exclusion criteria are shown in Table 2.

**Table 2:** Inclusion and exclusion criteria

Inclusion criteria	Exclusion criteria
Estimated age 18–80 years	Traumatic cardiac arrest (including strangulation, electrocution, and patients rescued from avalanches)
Out-of-hospital cardiac arrest	Accidental hypothermia with temperature < 32 °C
Non-traumatic cardiac arrest	Suspected non-traumatic haemorrhage as aetiology of the arrest
Less than 10 min from debut of arrest to start of basic or advanced cardiac life support	Pregnancy (obvious or suspected)
ACLS is established and can be continued	Suspected cerebral haemorrhage as aetiology of the arrest
	Patient included to the study site’s E-CPR protocol
	Other factors as decided by the treatment team (environmental factors, safety factors, and others)

ACLS, advanced cardiovascular life support; E-CPR, extracorporeal cardiopulmonary resuscitation

### Allocation and randomisation

**Fig. 1:**
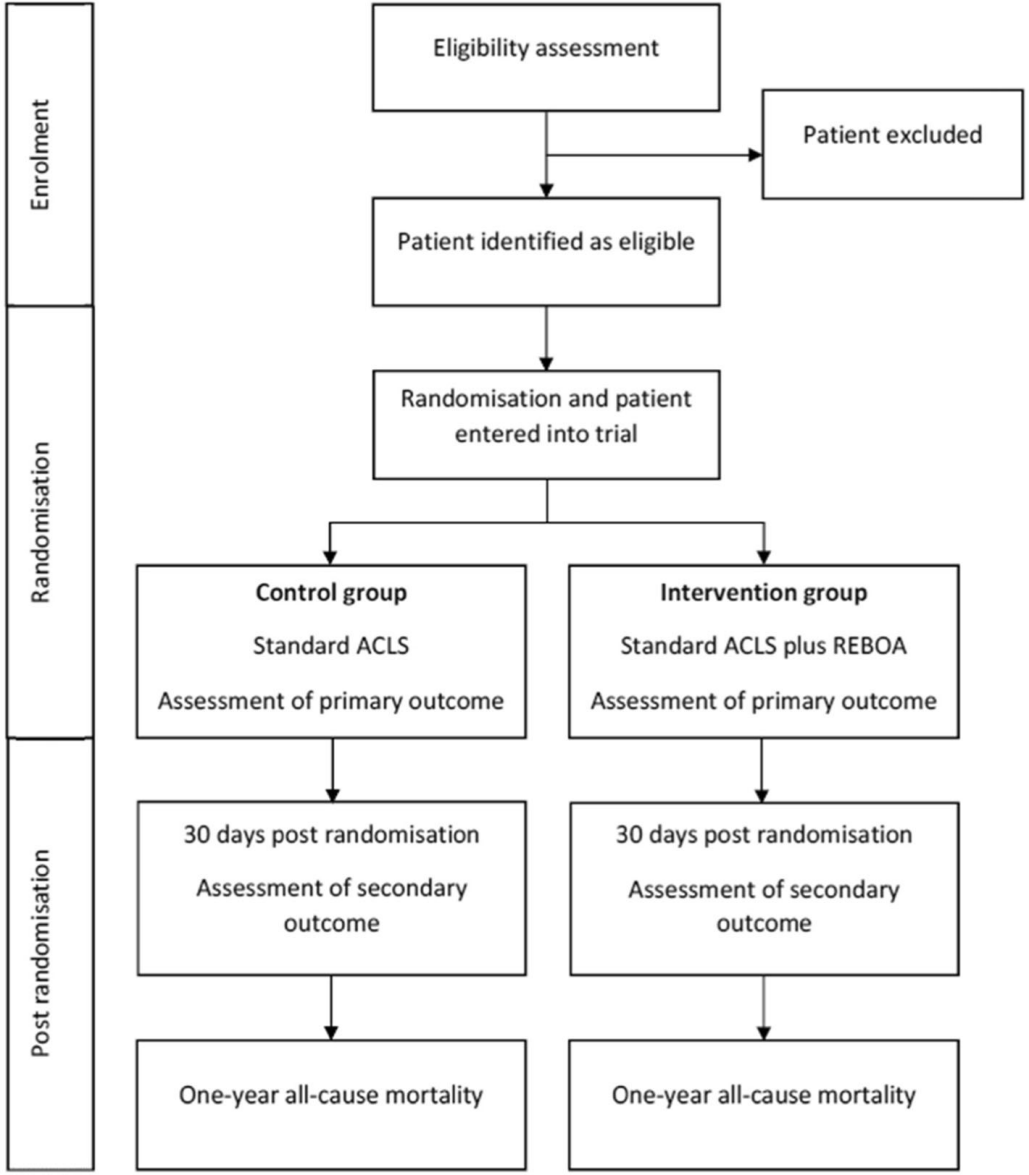
Flow chart showing the patient allocation process. ACLS, advanced cardiovascular life support; REBOA, resuscitative endovascular balloon occlusion of the aorta

Patients will be allocated in a 1:1 ratio between the two study arms (Fig. 1), according to the intention to treat principle. A permuted block randomisation method stratified by site will be used to allocate eligible patients to either the control group or the intervention group. Sealed envelopes allocating patients will be opened on-scene when a patient is eligible for randomisation. The randomisation lists will be produced by KlinForsk [30]. The random allocation sequence is generated by KlinForsk and no investigator has access to the allocation sequence.

### Interventions

All patients, regardless of randomisation group, will receive ACLS as described in the guidelines published by the ERC [5], Norwegian Resuscitation Council [4], and other local national guidelines. Both manual compressions and a mechanical chest compression machine are allowed. Airway management includes either endotracheal intubation or a supraglottic airway device

#### Intervention group

The intervention group will receive the same ACLS treatment as the control group. This group will also receive the intervention (REBOA) as adjunctive treatment. Additionally, intravenous or intraosseous access must be established via the upper body. Two types of REBOA catheter will be used in the trial: the REBOA Medical 20-mm balloon (REBOA Medical AS, Asker, Norway) and the ER-REBOA catheter (Prytime Medical Devices Inc., Boerne, TX, USA). Other catheters may be available during the trial period and will be considered for use. All catheters will be used according to their medical device approval. The use and insertion will follow the procedure described by the manufacturer.

### Outline of the REBOA procedure

A detailed description of the procedure is found in the study protocol, which is available as supplemental material and on the trial website (www.reboarrest.com).

**Fig. 2:**
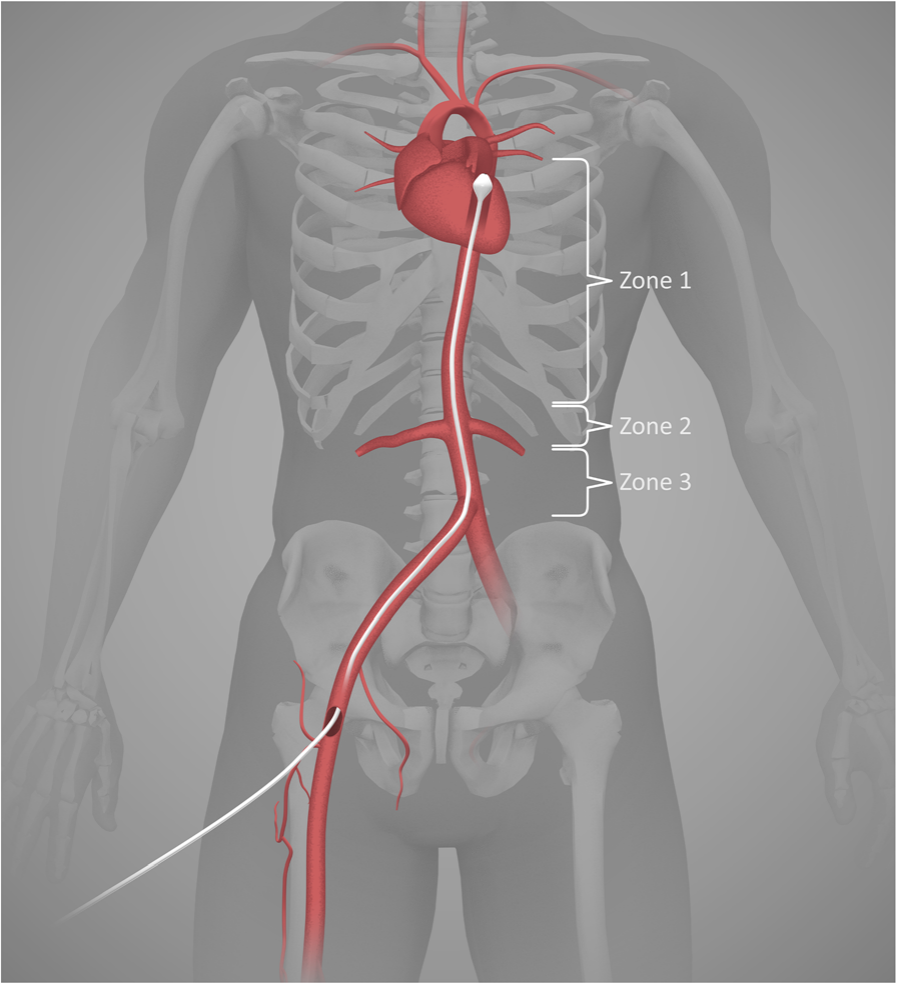
Illustration showing a REBOA balloon occluding the aorta in zone 1 after insertion of the catheter through the right femoral artery. REBOA, resuscitative endovascular balloon occlusion of the aorta

Antiseptic wash with chlorhexidine or similar solution will be performed before cannulation of the femoral artery. This will be performed under ultrasound guidance using an out-of-plane technique with insertion of a flexible guidewire. The cannulation can be performed during the ventilation phase of CPR or during a 10-20 s pause between chest compressions. After the guidewire is placed, ultrasound images of the guidewire position will be obtained and stored. A 7-Fr introducer sheath will be inserted over the guidewire and the stylet removed. A balloon sheath will be inserted through the introducer and placed at 50 cm for a zone 1 aortic occlusion [31] (Fig. 2). Zone 1 was chosen because it has the best possible haemodynamic effect [31, 32].

If feasible, arterial pressure measurements will be recorded from the distal tip of the catheter. After insertion and before the balloon is inflated, the left radial artery will be checked for a palpable pulse. The balloon will then be inflated with sterile 0.9% sodium chloride solution, and the radial pulse check will be repeated. If a palpable pulse is present, the location of the balloon will be accepted. If a previously present pulse disappears after balloon inflation, the balloon will be deflated, the sheath withdrawn by 5 cm, and the inflation/pulse check will be repeated. The duration of resuscitation effort will be as per the standard routine, regardless of whether REBOA is used. If ROSC is achieved, the balloon will be slowly deflated over 30 s and left in situ. Post-ROSC treatment will then be administered as per the standard routine.

### Sample size

**Fig. 3:**
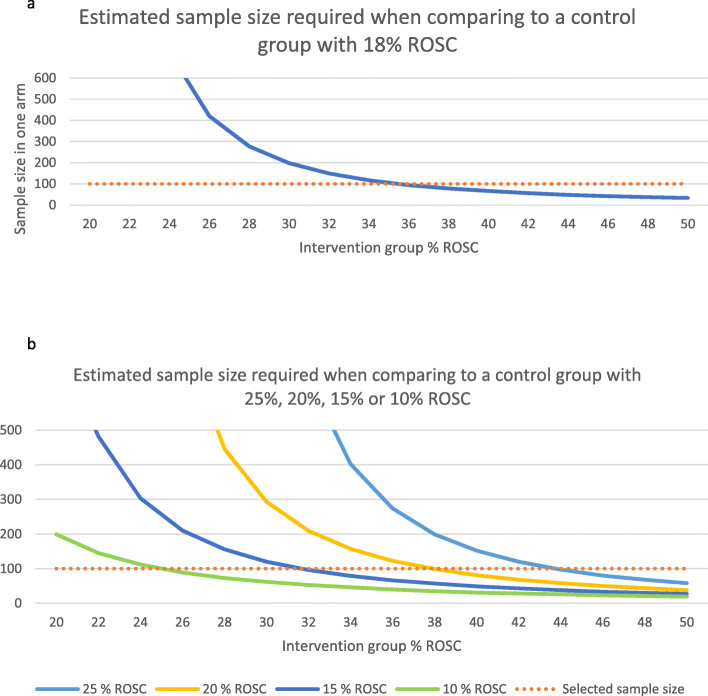
**a** Estimated sample size with return of spontaneous circulation as the primary endpoint. A horizontal line is drawn at the selected sample size of 100. ROSC, return of spontaneous circulation. **b** Estimated sample size with return of spontaneous circulation as the primary endpoint, compared to a control group with 25%, 20%, 15%, or 10% rate of ROSC. A horizontal line is drawn at the selected sample size of 100. ROSC, return of spontaneous circulation

Data to support sample size estimation for this study are scarce. A previous prospective study of REBOA in OHCA was an uncontrolled pilot study that included 10 patients, six of whom achieved ROSC [26]. In Norway, the overall ROSC rate is 32% [1]; however, the ROSC rate is reported to be only 18% in the patients with cardiac arrest who would meet the inclusion criteria for this trial [33]. We consider an increase in ROSC from 18 to 36% to be clinically relevant. The sample size needed to demonstrate this with 0.80 power and a significance level of 0.05 was calculated to be 94 patients in each group [34] (Fig. 3a). The sample size is first estimated as there were no interim analyses, and then corrected by using an inflation factor to account for the interim analyses. To account for dropouts, the sample size has been set to 100 patients per arm. The assumption of 18% rate of ROSC in the control group is uncertain and any deviation from this will impact the sample size estimations (Fig. 3b).

### Trial oversight

#### Data monitoring committee

An independent data monitoring committee (DMC) will be established and function as the safety board, governed by its own charter. The DMC will perform the interim analyses, review recruitment, data quality, protocol deviations, safety, and adverse events at set intervals.

#### Case review panel

A case review panel consisting of an interventional radiologist, a cardiologist, and HEMS physician will have access to all data from the resuscitation (de-identified). This includes patient monitor and defibrillator files, journal written by ambulance, and air ambulance crew and will report to the project management and the DMC on safety issues. The case review panel will assess the quality of the ACLS, with regard to duration of hands-off time, correct depth of compressions, adequate defibrillations and medications, and correct arterial placement of the guidewire.

#### Monitoring

A risk-based data monitoring procedure will be in place. This allows for clinical trial monitoring by KlinForsk that fulfils all regulatory requirements and ICH–GCP guidelines without the need for 100% source verification of the patient data. The monitoring procedure includes performing a risk analysis to identify high-risk elements of the study concerning patient safety and the primary endpoint data.

#### Adverse events and device effects

Adverse events and device effects follow ISO 14155:2020 Clinical investigation of medical devices for human subjects — Good clinical practice. Each adverse event will be assessed for seriousness, causality, severity, and expectedness. For each patient, the standard period for collecting and recording adverse events will be from randomisation and until admission to hospital, with an extension up to the 30-day follow-up visit for serious adverse events. An extensive list of expected adverse events both due to ACLS and REBOA is available in the detailed study protocol (supplemental material).

#### Summary of activities

The SPIRIT schedule (Table 3) provides a summary of activities and timeline for participants in the trial. In addition to comprehensive data capture during the pre-hospital phase for the primary objective of the trial, the study collects data on all participants admitted to hospital to assess secondary and explorative endpoints.

**Table 3:** SPIRIT schedule with study process and data collection during study period

	Study period					
	Enrolment	Randomisation	Post randomisation			
Timepoint	-***T***	***T*** = 0	Hospitalisation	***T*** < week 2	***T*** + 30 day	***T*** + 1 year
**Enrolment**						
Inclusion criteria	X					
Exclusion criteria	X					
Allocation		X				
Informed consent			X	X		
**Interventions**						
Intervention or control group		X				
**Assessments**						
ROSC		X				
Survival		X	X	X	X	X
**Pre-hospital registrations**						
Utstein styled documentation	X	X				
End-tidal CO_2_		X				
REBOA-procedure related data		X				
All relevant dispatch/procedure times	X	X	X			
**In-hospital registrations**						
Blood gas from admission			X			
Blood sample analysis			X	X		
Urine output			X	X		
Complications related to REBOA			X	X	X	
Cardiac interventions			X	X	X	
Length of stay intensive care			X	X	X	
Length of stay hospital			X	X	X	
Length of invasive respiratory support			X	X	X	
Length of renal replacement therapy			X	X	X	
Modified Rankin scale					X	
Adverse events		X	X	X	X	

SPIRIT, Standard Protocol Items: Recommendations for Interventional Trials; ROSC, return of spontaneous circulation; REBOA, resuscitative endovascular balloon occlusion of the aorta

### Statistical methods

A separate detailed statistical analysis plan will be developed in cooperation with the study statisticians and published before the first interim analysis.

The documentation on effect of REBOA in non-traumatic cardiac arrest is scarce, and the assumptions made when planning this study are uncertain. Therefore, a group sequential design with adaptive sample size modification has been chosen. This will allow (1) the possibility of stopping the trial early if significant between-group differences in either the primary endpoint or 30-day survival rate with good neurological outcome are found and (2) re-estimation of the sample size at the interim analyses to maintain the desired statistical power. An adaptive design has been advocated and is used in clinical research for several reasons [35–38], including cost-effectiveness, potential need for fewer study participants, ethical arguments concerning the safety and efficacy of the trial intervention, and the possibility of mimicking real-life medical practice more than a traditional randomised controlled trial.

The primary analysis will be conducted according to the intention-to-treat principle to compare the outcome between all participants randomised to the control group and the intervention group, that is, all patients in the REBOA group will be included in the analysis regardless of actual occlusion of the aorta. Per-protocol analyses will be considered if a considerable proportion of the REBOA group deviate from the protocol, thereby undermining the validity of the intention-to-treat analysis. Deviation from the protocol could be a consequence of the patient either achieving ROSC or being declared dead prior to aortic occlusion or from the procedure being aborted for whatever reason.

The primary endpoint and other dichotomous secondary endpoints will be analysed by logistic regression or hypothesis testing. The choice of test will depend on the need to adjust for covariates and the success rate. Continuous secondary endpoints (end-tidal CO_2_ values and blood pressure) will be analysed by regression methods.

#### Interim analyses

The DMC will perform three interim analyses, after 30, 60, and 90 patients are included in each of the study groups. These interim analyses will inflate the α (type I error rate). To control this inflation and keep the overall significance level of 0.05, the O’Brien-Fleming boundaries are used [39, 40]. This approach allows not to burn the whole *α* before the final analysis, which will be tested against a significance level of 0.043.

The primary endpoint and the secondary endpoint “30-day survival rate with good neurological status, defined as a modified Rankin scale score of 0–3” will be assessed in the interim analyses. The statistician will be blinded. The DMC will consider recommending the sponsor to stop the trial after an interim analysis if there is a significant difference in either the primary endpoint or the secondary endpoint “30-day survival rate with good neurological status, defined as a modified Rankin scale score of 0–3” between the groups.

At the second interim analysis, the DMC will perform a sample size calculation based on the assumption that the current difference in the primary endpoint between the two groups will persist. If the sample size needed to confirm a difference is more than three times the planned sample size (with 0.80 power and a significance level of 0.05), the DMC will consider recommending to the sponsor that the trial be stopped due to futility.

At the last interim analysis, the DMC will re-estimate the sample size in the event of a non-significant difference in the primary endpoint [41]. If the estimated sample size needed to confirm a difference between the groups is > 100 but ≤ 150 in each group (with 0.80 power and a significance level of 0.05), the final sample size will be modified, and the DMC would recommend the sponsor to continue the trial until the modified sample size is reached.

## Discussion

To perform a clinical trial in patients with critical illness in the pre-hospital environment is challenging. The balance between patient safety and autonomy and the need to ensure the safety and efficacy of medical interventions prompts the need to conduct well designed randomised trials. The present study is designed to balance these concerns. Informed consent prior to randomisation is impossible in unconscious patients. REBOARREST have received ethical approval to include patients with deferred consent. Next-of-kin will be asked for deferred consent after a patient is admitted to hospital or declared dead at the scene. Patients who regain the capacity to provide informed consent within 3 months will be asked for renewed deferred consent. Patients may withdraw from the study at any time without the need for a rationale and without compromising their medical care. If the patient has not regained ability to provide consent, the next-of-kin can withdraw the patient from the study at any time. Data on adverse events registered for patients who withdraw from the study will be stored in the database in an anonymized form to ensure that no safety information is lost.

The use of REBOA in non-traumatic OHCA is a novel adjunct to resuscitation efforts. However, given the results emerging from preclinical studies and human reports, we believe that there is a need for a large-scale trial to investigate the safety and efficacy of REBOA as an adjunctive treatment in patients with non-traumatic OHCA.

## Trial status

The trial is ongoing and patient recruitment started 15 June 2021. We estimate a 3-year recruitment period; hence, the estimated date for completed recruitment is June 2024. Study protocol version 1.3 dated 14 June 2021 is available as a supplemental material.

## Supplementary Information

**Additional file 1:.** SPIRIT 2013 Checklist: Recommended items to address in a clinical trial protocol and related documents

**Additional file 2:.** The REBOARREST trial

## Data Availability

The datasets generated and/or analysed during the current study are available from the corresponding author on reasonable request.

## Abbreviations

ACLS: Advanced cardiovascular life support
CPR: Cardiopulmonary resuscitation
DMC: Data monitoring committee
ERC: European Resuscitation Council
ICH-GCP: International Council for Harmonization of Technical Requirements – Good Clinical Practice
OHCA: Out-of-hospital cardiac arrest
REBOA: Resuscitative endovascular balloon occlusion of the aorta
ROSC: Return of spontaneous circulation
