# Supplementary material for: REBOARREST, resuscitative endovascular balloon occlusion of the aorta in non-traumatic out-of-hospital cardiac arrest: a study protocol for a randomised, parallel group, clinical multicentre trial
Source: Trials. 2021 Jul 31;22:511. doi: 10.1186/s13063-021-05477-1 (PMC8325811; doi:10.1186/s13063-021-05477-1)
Supplement: Supplementary file 2 — Additional file 2:. The REBOARREST trial [file 13063_2021_5477_MOESM2_ESM.docx]

**The REBOARREST trial**

**A randomised trial on resuscitative endovascular balloon occlusion of the aorta in non-traumatic out of hospital cardiac arrest**

The Universal Trial Number (UTN) WHO: U1111-1253-0322

**Sponsor: Clinic of Cardiology, St. Olavs University Hospital**

**Funder: Norwegian Air Ambulance Foundation**

**Chief Investigator: Dr. Andreas Jørstad Krüger, MD, PhD**

**Project Manager: Dr. Jostein Rødseth Brede, MD**

Protocol version 1.3 Date 14.06.2021

**Revision history**

| **Version number**  **Date** | **Description** | **Date approved REC** |
| --- | --- | --- |
| 1.0  09.06.2020 | Original submission |  |
| 1.1  09.09.2020 | New version number and date  Change of data retention period from 10 to five years and total study period from 15 to 10 years. | 28.09.2020 |
| 1.2  01.02.2021 | New version number and date  Change in the text in **6.3 The primary endpoint.** Specification and detailed description are added  Change/specification in **7.2 Inclusion criteria**, removed “*Witnessed arrest or*” and “*Commenced*” from third and fourth inclusion criteria.  Change/specification in **7.3 Exclusion criteria**:   - Removed “*Age less than 18 years or above 80 years*” - Specified to “*Accidental hypothermia with temperature < 32 ^0^C”* - Added “*Suspected non-traumatic haemorrhage as aetiology of the arrest*”   Added “*Asphyxia as probable cause of arrest*” in **9.8 Specification of exploratory subgroups**  Edited **10.1 Randomisation procedure,** change from “*one randomised block*” to “*envelopes*”  Added cerebral oximetry equipment to **10.2.1 Medical devices in both groups** and **11.1.2 During treatment** | 11.02.2021 |
| 1.3  14.06.2021 | New version number and date  Change in text in **6.5 Exploratory endpoints**, to “*Incidence of all adverse events*” |  |

# Contact details

**Sponsor**

Name Clinic of Cardiology, St. Olavs University Hospital

Contact person Rune Wiseth, Head of Clinic

Address Postboks 3250 Torgarden, 7006 Trondheim, Norway

E-mail address [post.hjertemedisin@stolav.no](mailto:post.hjertemedisin@stolav.no)

**Chief Investigator**

Name Andreas Jørstad Krüger

Professional position MD, PhD, Consultant Anaesthesiologist

Address Luftambulansebasen Trondheim, Vestre Rosten 114, 7075 Trondheim, Norway.

E-mail address [Andreas.kruger@ntnu.no](mailto:Andreas.kruger@ntnu.no)

Phone number +47 908 62 586

**Project Manager**

Name Jostein Rødseth Brede

Professional position MD, Consultant Anaesthesiologist

Address Postboks 3250 Torgarden, 7006 Trondheim, Norway.

E-mail address [Jostein.brede@norskluftambulanse.no](mailto:Jostein.brede@norskluftambulanse.no)

Phone number +47 994 45 914

**Main funder**

Name Norwegian Air Ambulance Foundation

Contact person Tom Hanssen, Director of Research and Development

E-mail address [tom.hanssen@norskluftambulanse.no](mailto:tom.hanssen@norskluftambulanse.no)

## Project management

**(PM) Jostein Rødseth Brede**, MD ^1^ Department of Emergency Medicine and Pre-hospital Services, St. Olavs University Hospital, Trondheim, Norway

^2^ Department of Anaesthesiology and Intensive care, St. Olavs University Hospital, Trondheim, Norway

^3^ Department of Research, Norwegian Air Ambulance Foundation, Oslo, Norway

^4^ Department of Circulation and Medical Imaging, Faculty of Medicine and Health Sciences, Norwegian University of Science and Technology (NTNU), Trondheim, Norway

**(CI) Andreas Jørstad Krüger**, MD, PhD ^1^ Department of Emergency Medicine and Pre-hospital Services, St. Olavs University Hospital, Trondheim, Norway

^3^ Department of Research, Norwegian Air Ambulance Foundation, Oslo, Norway

^4^ Department of Circulation and Medical Imaging, Faculty of Medicine and Health Sciences, Norwegian University of Science and Technology (NTNU), Trondheim, Norway

**Marius Rehn**, MD, PhD ^3^ Department of Research, Norwegian Air Ambulance Foundation, Oslo, Norway

^5^ Air Ambulance Department, Division of Prehospital Services, Oslo University Hospital, Oslo, Norway

^6^ Faculty of Health Sciences, University of Stavanger, Stavanger, Norway

**Jostein Dale**, MD ^1^ Department of Emergency Medicine and Pre-hospital Services, St. Olavs University Hospital, Trondheim, Norway

**Arne Kristian Skulberg,** MD, PhD ^3^ Department of Research, Norwegian Air Ambulance Foundation, Oslo, Norway

^5^ Air Ambulance Department, Division of Prehospital Services, Oslo University Hospital, Oslo, Norway

**Kjetil Thorsen,** PhD ^3^ Department of Research, Norwegian Air Ambulance Foundation, Oslo, Norway

PM – Project Manager

CI – Chief Investigator

# Signatures

**Sponsor signatory approval**

I hereby declare that I will conduct the study in compliance with the protocol and the applicable regulatory requirements:

Name: Rune Wiseth

------------------------------------------------------------------------------------------------------- -----------------------------

Sponsor signature Date

**Chief Investigator signatory approval**

I hereby declare that I will conduct the study in compliance with the protocol and the applicable regulatory requirements. This document contains confidential and proprietary information and may not be copied or reproduced in whole or part without the written permission of sponsor. I agree to conduct in person or to supervise the study. I agree to ensure that all who assist me in the conduct of the study have access to the research protocol plus any amendments and are aware of their obligations.

Name: Andreas Jørstad Krüger

------------------------------------------------------------------------------------------------------- -----------------------------

Chief Investigator signature Date

**Other signatures**

I hereby declare that I will conduct the study in compliance with the protocol and the applicable regulatory requirements and study documents:

Name Role Signature Date

Jostein Rødseth Brede Project manager

Kjetil Thorsen Study statistician

# Clinical study summary

**Title**: Resuscitative endovascular balloon occlusion of the aorta in non-traumatic out of hospital cardiac arrest – The REBOARREST Trial

**Study objectives**: The primary objective is to assess the efficacy of resuscitative endovascular balloon occlusion of the aorta (REBOA) as an adjunct treatment to advanced cardiovascular life support (ACLS) in patients with out-of-hospital cardiac arrest.

Secondary objectives are 1) to describe the hemodynamic physiology of aortic occlusion during ACLS, 2) a description of any adverse events and 3) description of 30-days survival.

**Clinical study design**: A prospective, randomised, parallel group, multi-centre, phase II clinical trial. Patients are randomised in a 1:1 ratio to be included to the control group or the intervention group. 200 patients will be included in the trial.

**Intervention and control**: The control group receives ACLS according to national guidelines.

The intervention group receives ACLS according to national guidelines and the REBOA procedure as an adjunct treatment.

**Inclusion criteria**: Age 18 to 80 years, out-of-hospital cardiac arrest, non-traumatic cardiac arrest, witnessed arrest or less than 10 minutes from debut of arrest to start of basic or advanced cardiac life support, commenced ACLS is established and can be continued.

**Exclusion criteria**: Age less than 18 or above 80 years, traumatic cardiac arrest, hypothermia, pregnancy, suspected cerebral haemorrhage as aetiology of the arrest, unable to perform ACLS as per national guideline, patient included to the study site’s E-CPR protocol, other factors as decided by the treatment team (environmental factors, safety factors and others).

**Primary endpoint**: The primary endpoint of this study is the proportion of patients that achieve return of spontaneous circulation (ROSC).

**Secondary endpoints**: -30-day survival rate with good neurologic status defined as modified Rankin scale (mRS) score 0-3.

-Difference in end-tidal CO2 measurements in the control group and the intervention group after aortic occlusion.

-Change in blood pressures after aortic occlusion.

-Difference in left ventricular ejection fraction measured by echocardiography

**Safety reporting**: -A three-step safety program will be conducted: 1) After each included patient, the physician will be interviewed by an investigator. 2) Ultrasound images from patients in the intervention group will be reviewed to ensure arterial catheter placement. 3) An external expert panel will perform a case review using all available information.

-Serious adverse events and serious adverse device events will be reported.

-An independent data monitoring committee will be established.

**Duration of study**: The trial period for each patient is one year after randomisation. The trial is expected to start winter 2020/2021. Inclusion of patients is estimated to take three years. Follow up and five-year data retention period necessitate a total study period of 10 year.

**Follow up**: Patient journal will be examined one year after randomisation to assess survival.

[1 Contact details 3](#_Toc42542846)

[1.1 Project management 4](#_Toc42542847)

[2 Signatures 5](#_Toc42542848)

[3 Clinical study summary 6](#_Toc42542849)

[4 Abbreviations 10](#_Toc42542850)

[5 Introduction 11](#_Toc42542851)

[5.1 Background 11](#_Toc42542852)

[5.1.1 Current knowledge and practice 11](#_Toc42542853)

[5.1.2 Other adjuncts to advanced cardiovascular life support 12](#_Toc42542854)

[5.1.3 Rationale for use and anticipated clinical benefits of REBOA 12](#_Toc42542855)

[5.1.4 Epidemiology of cardiac arrest 13](#_Toc42542856)

[5.2 Study Rationale 13](#_Toc42542857)

[5.3 Academic study 14](#_Toc42542858)

[6 Objectives and endpoints 14](#_Toc42542859)

[6.1 Primary objective of the study 14](#_Toc42542860)

[6.2 Secondary objective of the study 14](#_Toc42542861)

[6.3 The primary endpoint 14](#_Toc42542862)

[6.4 The secondary endpoints 14](#_Toc42542863)

[6.5 Exploratory endpoints 15](#_Toc42542864)

[7 Study population 15](#_Toc42542865)

[7.1 Selection of participants and study setting: 15](#_Toc42542866)

[7.2 Inclusion criteria 15](#_Toc42542867)

[7.3 Exclusion criteria 16](#_Toc42542868)

[7.4 Criteria for withdrawal or discontinuation 16](#_Toc42542869)

[7.5 Enrolment 16](#_Toc42542870)

[8 Informed consent process 16](#_Toc42542871)

[9 Statistics 17](#_Toc42542872)

[9.1 Statistical hypothesis 17](#_Toc42542873)

[9.2 Sample size 17](#_Toc42542874)

[9.3 Interim analyses 18](#_Toc42542875)

[9.4 Measures to minimize bias 19](#_Toc42542876)

[9.5 Statistical design 19](#_Toc42542877)

[9.6 The level of significance and power of the clinical study 19](#_Toc42542878)

[9.7 Expected drop out rates 20](#_Toc42542879)

[9.8 Specification of exploratory subgroups 20](#_Toc42542880)

[9.9 Treatment of missing data 20](#_Toc42542881)

[9.10 Min/max number of subjects per centre 20](#_Toc42542882)

[10 Project methodology and overall design 20](#_Toc42542883)

[10.1 Randomisation procedure 21](#_Toc42542884)

[10.2 Medical devices in the study 22](#_Toc42542885)

[10.2.1 Medical devices in both groups 22](#_Toc42542886)

[10.2.2 Medical devices in the intervention group only 22](#_Toc42542887)

[10.2.3 Kit for REBOA intervention 23](#_Toc42542888)

[10.3 Expected duration 23](#_Toc42542889)

[11 Study procedures 23](#_Toc42542890)

[11.1 By Visit 24](#_Toc42542891)

[11.1.1 Before Treatment Starts 24](#_Toc42542892)

[11.1.2 During Treatment 24](#_Toc42542893)

[11.1.3 End of Study Visit 27](#_Toc42542894)

[11.2 After End of Treatment (Follow-up) 27](#_Toc42542895)

[11.2.1 Follow-up medical care 27](#_Toc42542896)

[11.3 Trial Discontinuation 27](#_Toc42542897)

[11.4 Laboratory Tests 28](#_Toc42542898)

[11.5 Concomitant therapy 28](#_Toc42542899)

[12 Data collection and processing 28](#_Toc42542900)

[12.1 Data handling 28](#_Toc42542901)

[12.2 Data collection during study period 29](#_Toc42542902)

[12.3 Procedures for data review and editing 33](#_Toc42542903)

[12.4 Specified retention period 34](#_Toc42542904)

[13 Safety assessment 34](#_Toc42542905)

[13.1 Risks and benefit 34](#_Toc42542906)

[13.1.1 Specific risk for interruption of ACLS quality 34](#_Toc42542907)

[13.1.2 Specific risk of venous placement of REBOA catheter 34](#_Toc42542908)

[13.1.3 Other risks 35](#_Toc42542909)

[13.1.4 Autopsy 36](#_Toc42542910)

[13.1.5 Steps to be taken to control or mitigate risks 37](#_Toc42542911)

[13.2 Summary of risk assessment 37](#_Toc42542912)

[14 Adverse events 37](#_Toc42542913)

[14.1 Definitions of adverse events and device deficiencies 38](#_Toc42542914)

[14.2 Adverse events in the REBOARREST trial 39](#_Toc42542915)

[14.3 Assessment of seriousness 40](#_Toc42542916)

[14.4 Assessment of causality 40](#_Toc42542917)

[14.5 Assessment of severity 41](#_Toc42542918)

[14.6 Assessment of expectedness 41](#_Toc42542919)

[14.7 Period for recording adverse events 43](#_Toc42542920)

[14.8 Recording of adverse events 43](#_Toc42542921)

[14.9 Reporting of SAE/SADE/USADE 44](#_Toc42542922)

[14.9.1 Reporting from study site to chief investigator 44](#_Toc42542923)

[14.9.2 Reporting to appropriate medicinal authorities 44](#_Toc42542924)

[14.10 SAE/SADE/USADE advice 45](#_Toc42542925)

[15 Study management 45](#_Toc42542926)

[16 Study Amendments 45](#_Toc42542927)

[17 Ethics Committee Approval 45](#_Toc42542928)

[18 Other Regulatory Approvals 45](#_Toc42542929)

[19 Trial insurance 46](#_Toc42542930)

[20 Trial organisation 46](#_Toc42542931)

[20.1 Statement that investigator is not allowed to deviate from the research protocol 47](#_Toc42542932)

[20.2 Procedures for recording, reporting and analysing protocol deviations 47](#_Toc42542933)

[20.3 Trial Master File 48](#_Toc42542934)

[21 Study administration structure 48](#_Toc42542935)

[21.1 Research responsible 48](#_Toc42542936)

[21.2 Project management group 48](#_Toc42542937)

[21.3 Cooperating institutions and study sites 48](#_Toc42542938)

[22 Data monitoring committee 49](#_Toc42542939)

[23 Monitoring 49](#_Toc42542940)

[24 Funding 49](#_Toc42542941)

[25 Conflict of Interest 50](#_Toc42542942)

[26 Data Sharing 50](#_Toc42542943)

[27 Publication policy 50](#_Toc42542944)

[28 Bibliography 52](#_Toc42542945)

[29 Appendices 57](#_Toc42542946)

[29.1 SPIRIT checklist 57](#_Toc42542947)

[29.2 The modified Rankin scale 57](#_Toc42542948)

[29.3 REBOA catheters product information and instruction for use 57](#_Toc42542949)

[29.4 Label for REBOA procedure kit 57](#_Toc42542950)

[29.5 On-scene checklist for pre-hospital physician 57](#_Toc42542951)

[29.6 Interview template 57](#_Toc42542952)

[29.7 Subject ID log 57](#_Toc42542953)

[29.8 Screening log 58](#_Toc42542954)

[29.9 Delegation log 58](#_Toc42542955)

# Abbreviations

ACLS Advanced Cardiovascular Life Support

ADE Adverse Device Effects

AE Adverse Event

CA Cardiac Arrest

CPR Cardiopulmonary Resuscitation

CRF Case Report Form

DMC Data monitoring committee

ECMO Extra-Corporeal Membrane Oxygenation

E-CPR Extracorporeal Cardiopulmonary Resuscitation

EtCO2 End Tidal CO2

HEMS Helicopter Emergency Medical Services

LVEF Left Ventricular Ejection Fraction

mRS Modified Rankin Scale

OHCA Out of Hospital Cardiac Arrest

PCI Percutaneous Coronary Intervention

P-EMS Physician-staffed Emergency Medical Services

REBOA Resuscitative Endovascular Balloon Occlusion of the Aorta

REC Regional Ethics Committee

ROSC Return of Spontaneous Circulation

SADE Serious Adverse Device Effect

SAE Serious Adverse Event

SI Site Investigator and/or designee

SPIRIT Standard Protocol Items: Recommendations for Interventional Trials

USADE Unanticipated Serious Adverse Device Effect

# Introduction

This is a randomised, parallel group, multi-centre, phase II clinical trial designed to assess the efficacy and hemodynamic effect of resuscitative endovascular balloon occlusion of the aorta (REBOA) in study subjects suffering from non-traumatic out of hospital cardiac arrest (OHCA).

200 patients will be enrolled in this study, 100 patients in each group.

This is the first prospective trial in the world to assess the efficacy of REBOA in non-traumatic cardiac arrest. The intervention is shown feasible in the pre-hospital setting^1^. If this trial provides a signal of benefit in patients, this study could initiate further clinical research which could change current resuscitation practice world-wide.

This is a multi-centre trial, with centres being consecutively recruited. Centres may include helicopter and/or rapid response car. Identified potentially centres are:

- Norwegian Air Ambulance base at Rosten, Trondheim, Norway.
- Rescue Helicopter Service at Ørland Main Airforce base, Brekstad, Norway.
- Norwegian Air Ambulance base in Bergen, Norway.
- Department of Air Ambulance Services, Prehospital Division, Oslo University Hospital, Norway.

Additional study sites may be eligible later.

This protocol is designed according to the Standard Protocol Items: Recommendations for Interventional Trials (SPIRIT) 2013 guidelines (appendix 29.1) and will be reported according to the Consolidated Standards Of Reporting Trials 2010 Guidelines ^2,3^.

## Background

### Current knowledge and practice

OHCA carries a high mortality rate, with 30-day survival rate of 14% in Norway ^4^. For non-traumatic cardiac arrest, the most frequent aetiology is a cardiac disease ^5,6^. Cardiopulmonary resuscitation (CPR) prolongs the time prior to hypoxic irreversible damage, by delivery of partially oxygenated blood to vital organs ^7^. The brain is sensitive to hypoxia and the high mortality rate after the patients are admitted to hospital is primarily caused by anoxic brain damage ^8^.

The treatment of out-of-hospital cardiac arrest are advanced cardiovascular life support (ACLS) as stated in the guidelines from the Norwegian Resuscitation Council ^9^ and the European Resuscitation Guidelines ^10^ (Figure 1).


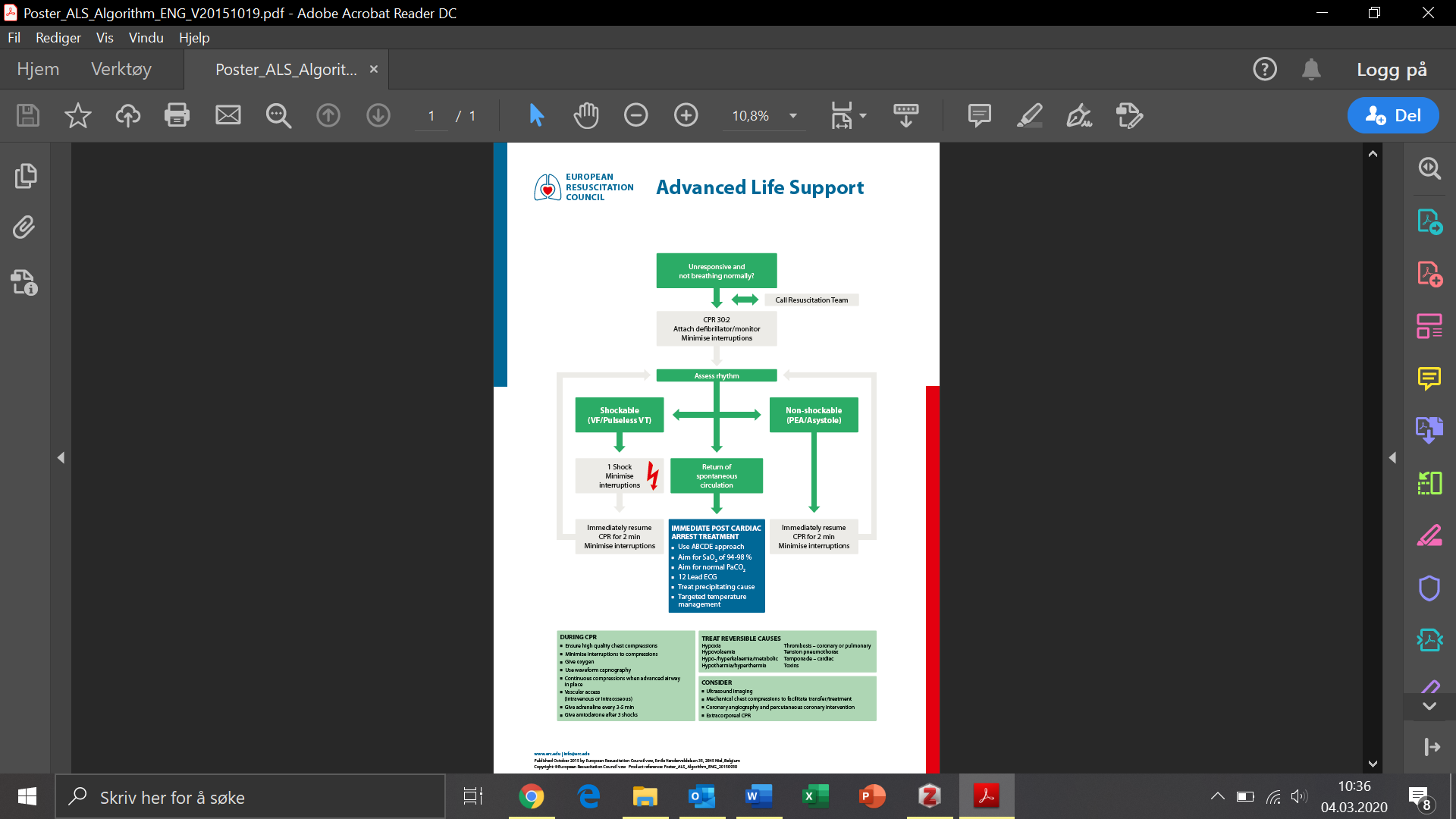


*Figure 1. Guidelines from the European Resuscitation Council*

### Other adjuncts to advanced cardiovascular life support

Other interventions such as fibrinolysis or percutaneous coronary intervention (PCI) are well documented therapies and recommended as appropriate ^10^. Extracorporeal membrane oxygenation (ECMO) is a possible intervention in the cardiac arrest setting ^11^. This is currently not recommended in the guidelines from the Norwegian Resuscitation Council but is in use in some centres. Some study centres, for instance Oslo University Hospital, may have an extracorporeal-CPR (E-CPR) protocol in place. Another future intervention is the selective aortic arch perfusion technique, in which oxygenated blood is injected into the aorta under pressure, through a special designed catheter ^12,13^. This technique has yet to be tested in patients.

### Rationale for use and anticipated clinical benefits of REBOA

REBOA is a technique used to provide occlusion of the aorta by inflation of an intra-aortic balloon. REBOA can be used in management of haemorrhagic shock or cardiac arrest secondary to trauma. Recently, REBOA has been proposed as an adjunct treatment in management of non-traumatic cardiac arrest patients ^14,15^. Thoracic aortic occlusion provides a redistribution of the cardiac output to organs proximal to the occlusion through reduction of vascular volume where generated cardiac output is distributed. Preclinical studies demonstrate that REBOA during cardiopulmonary resuscitation provide both increased coronary artery blood flow and perfusion pressure and increased rates of return of spontaneous circulation (ROSC) ^12,13,16–21^. The coronary perfusion pressure is the driving force of blood through the coronary arteries, calculated as the diastolic aortic pressure minus the pressure in the right atrium. Hence, increased aortic diastolic blood pressure will increase the coronary perfusion pressure. This is shown to be associated with ROSC in humans ^22^. REBOA during experimental resuscitation also increase blood flow to the carotid arteries ^19,23^, cerebral arteries ^17,18,23–25^ and cerebral perfusion pressure ^17,18,23,26^. These findings support our hypothesis that patients with non-traumatic cardiac arrest might benefit from REBOA during resuscitation. A few published case reports demonstrates the effect of REBOA on humans ^27–30^. Currently, only one study reports the prospective use of REBOA in clinical use ^1^. This pilot trial demonstrated that pre-hospital REBOA procedure during resuscitation is feasible and did not negatively influence the quality of the ACLS.

We anticipate that the intervention will provide an improvement in both systolic and diastolic blood pressure. This could potentially improve perfusion of the brain and heart during CPR. Brain tissue is highly sensitive to hypoxemia and improved systolic blood pressure, with possible improved perfusion of the brain, is likely beneficial. The potential clinical benefit in this trial is therefore increased rate of ROSC and survival to hospital, possibly also improved 30-day survival rate.

Besides the potential benefit of REBOA for cerebral and coronary circulation during CPR, insertion of the catheter may have some other advantages in the post-ROSC phase. The CHEER trial demonstrated that patients can survive with good neurological outcomes after more than 30 min of pre-hospital resuscitation if they were given circulatory support in the form of extracorporeal membrane oxygenation (ECMO) and definitive intervention in hospital ^31^. The introducer sheath in the femoral artery used to establish REBOA can be used for quick access to ECMO or other endovascular interventions such as PCI, directly after admission.

### Epidemiology of cardiac arrest

The number of out-of-hospital cardiac arrest in Norway was 3 172 in 2017, which correlates to an incidence of 60/100 000 inhabitants per year, increasing from 53/100 000 in 2015 ^4^. Many patients with OHCA have little comorbidity and a potential for good long-term function ^32^. A needs assessment based on the Norwegian Cardiac Arrest Registry show that 240 OHCA patients each year in Norway could be eligible for the REBOA procedure ^33^. This needs assessment analysis is based on a national data set. However, many European countries have a similar population pattern, and many have similar physician-staffed emergency medical services (P-EMS). The results of this needs assessment are therefore likely transferable to other Nordic countries. In countries with more dense population, more patients could be eligible for the REBOA procedure due to shorter P-EMS response intervals.

## Study Rationale

The purpose of the study is to assess the efficacy of REBOA as an adjunct treatment to ACLS in non-traumatic cardiac arrest and the effects of this intervention on ROSC. This is not a trial to test any medical device, nor to compare the effect of different REBOA catheters.

The use of REBOA during cardiopulmonary resuscitation is a novel procedure, which is only prospectively investigated in one published study ^1^. The procedure is researched in preclinical setting and animal models ^12,13,16–21,23–26^, however, only a few case reports are published on the effect in humans ^27–30^. This trial will therefore fill a knowledge gap. The survival rate after OHCA is dismal and a large part of these patients have small comorbidity burden ^32^. A possible beneficial intervention might therefore improve both survival rate and quality of life in the survivors. This trial will be important to obtain new knowledge for health services globally. If the hypothesis is correct and REBOA improves survival and/or cerebral status of the survivors, the trial can contribute to initiate further research that improve the current best practice.

## Academic study

This study is not part of an application for regulatory approval of medical devices. It is not conducted to establish or verify the clinical benefits of a device as specified by its manufacturer. All catheters used for balloon inflation of the aorta is CE marked and are used within their regulatory approval. No device manufacturer is involved in the design of this trial, nor will any manufacturer have access to data or results other than those in the public domain. The sponsor retain ownership of all data and results generated through this trial. It is not designed to report to any Notified Body to alter CE-markings or CE-approvals or other regulatory applications for medical devices.

# Objectives and endpoints

## Primary objective of the study

The primary objective is to assess the efficacy of REBOA as an adjunct treatment to advanced cardiovascular life support in patients with out-of-hospital cardiac arrest, measured as primary endpoint: return of spontaneous circulation.

## Secondary objective of the study

Secondary objectives of this trial are to describe the hemodynamic physiology of aortic occlusion during ACLS and a description of any adverse events and 30 days survival with good neurological status.

## The primary endpoint

The primary endpoint of this study is the proportion of patients that achieve return of spontaneous circulation (ROSC) with a duration of at least 20 minutes, i.e. sustained ROSC.

This clinical endpoint is based on the following Utstein definition updated in 2019 ^34^: *“Sustained ROSC is deemed to have occurred when chest compressions are not required for 20 consecutive minutes and signs of circulation persist.*”

Whether or not a patient has obtained ROSC is a comprehensive clinical assessment performed at regular intervals during the provision of CPR. This assessment is made in patients with an organized rhythm on ECG during pauses in chest compressions. ROSC should be suspected if one or more of the following occur ^34^:
•    Signs of life, including coughing, movement and/or regular breathing
•    Palpable pulses in radial, carotid and/or femoral arteries
•    Invasive systolic blood pressure > 50 mmHg

The current 2015 guidelines from the European Resuscitation Council state that a rise in measured end-tidal CO2 values (EtCO2) during CPR is indicative of ROSC ^10^. Establishment of REBOA may also give a transient increase in EtCO2 ^1^. There are no current cut-off values for EtCO2 deemed appropriate to use in this setting to indicate whether ROSC has occurred or not. Any rise in EtCO2 should be clinically assessed in conjunction with other signs of circulation.

If the patient has shorter periods of ROSC and do not obtain a persisting circulation, the patient has not met the primary endpoint. In the documentation, ‘any ROSC’, should be stated in these instances.

## The secondary endpoints

1. 30-day survival rate with good neurologic status defined as modified Rankin scale (mRS) score 0-3.

We dichotomise the mRs into good (score 0-3) and poor (score 4-6) neurological state (appendix 29.2) to enable both binary statistical analysis and a reasonable clinical interpretation of trial results, as performed in other large cardiac arrest studies ^35^. The mRS is commonly used as a primary outcome scale for acute stroke trials ^36^, but is increasingly used also in cardiac arrest research and has been recommended in the International Liaison Committee on Resuscitation as part of the Utstein template ^37,38^. The outcome is also in accordance with recently published Core Outcome Set for Cardiac Arrest trials ^39^.

1. Difference in end-tidal CO2 measurements in the control group and the intervention group after aortic occlusion.

EtCO2 measurements is a proxy measurement of central circulation, shown feasible in this setting ^1^. This pilot study reported a significant increase in EtCO2 after 30, 60 and 90 seconds. This allows for comparison of the two groups, with a possible increase in central circulation as a hypothesized reason to an increased proportion of patients achieving ROSC. In the control group, EtCO2 is measured after airway management is completed. In the intervention group, EtCO2 is measured before balloon inflation and 30, 60 and 90 seconds after balloon inflation.

1. Change in blood pressures after aortic occlusion.

In the intervention group, intra-aortic blood pressure measurements from the REBOA catheter are recorded. This apply only to the catheters with CE/FDA approval for invasive blood pressure measurements and will therefore be assessed in a sub-group of the participants. Continuous registration of invasive blood pressure will start before aortic occlusion.

1. Left ventricular ejection fraction (LVEF) measured by echocardiography

## Exploratory endpoints

1. All-cause mortality one year after randomization
2. Difference in organ function, using the Acute Kidney Injury Network (AKIN) classification, liver function blood tests and others.
3. Incidence of all adverse events

# Study population

## Selection of participants and study setting:

The patient population is adult patients (18 to 80 years of age, as estimated by crew on-scene) that suffers from an out-of-hospital cardiac arrest with assumed non-traumatic origin, as determined by the physician on scene.

## Inclusion criteria

The subject must meet all the following criteria to be included in the trial:

- Estimated age between 18 to 80 years
- Out-of-hospital cardiac arrest
- Non-traumatic cardiac arrest
- Less than 10 minutes from debut of arrest to start of basic or advanced cardiac life support
- ACLS is established and can be continued

## Exclusion criteria

Fulfilling one criterion excludes the subject to be included in this trial.

- Traumatic cardiac arrest, including strangulation, electrocution and patients rescued from avalanches
- Accidental hypothermia with temperature < 32 ^0^C
- Suspected cerebral haemorrhage as aetiology of the arrest
- Suspected non-traumatic haemorrhage as aetiology of the arrest
- Pregnancy, obvious or suspected
- Patient included to the study site’s E-CPR protocol
- Other factors as decided by the treatment team (environmental factors, safety factors and others)

## Criteria for withdrawal or discontinuation

Patients can withdraw from the clinical study at any time without the need of a rationale and without compromise to their future medical care. If the patient has not regained ability to give consent, the next-of-kin can withdraw the patient from the study at any time. All patients will receive the same standard of care.

Any safety data on adverse events registered in patients that withdraw from the study will be stored in the database in an anonymized form, to ensure that no safety information is lost.

Patients that withdraw or are withdrawn from the study after randomisation will be replaced.

## Enrolment

Subjects with out-of-hospital cardiac arrest in the areas serviced by the study centres are candidates for enrolment into the study. Patients who develop cardiac arrest while being treated for other causes by ambulance personnel or the P-EMS crew are also eligible.

# Informed consent process

Because of the nature of the disease, patient in cardiac arrest cannot provide informed consent before randomisation and inclusion. Hence, the intervention will be performed before the patient or next-of-kin can provide informed consent or withdraw from the study.

According to regulations and as applied in on-going studies on cardiac arrest patients, the patient’s next-of-kin will be asked for an informed consent. During CPR an informed consent from the next-of-kin cannot be obtained, both due to that the crew are performing the CPR and because the next-of-kin is expected to be in a psychological state where it is not possible to process such information. The next-of-kin will therefore be asked for consent after the patients are admitted to the hospital or declared dead at scene. They will be contacted as soon as possible after the incident and all information will be given by a senior physician experienced in communication with next-of-kin to patients with critical illness. Patients regaining capacity to give an informed consent within 3 months will be asked for a deferred consent.

This process adheres to the ethical principles that have their origin in the Declaration of Helsinki. Prior to the start of this trial, the written informed consent form and any other information intended to be provided to the subjects has been approved by the regional ethical committee (REC).

The site investigator and/or designee (collectively named SI) will conduct the informed consent process of explaining the clinical study to the subject as well as providing the subject with a copy of the subject information sheet. The consent information will include all aspects of the clinical study that are relevant to the subject’s decision to participate in the language in which the subject is most proficient. The language will be non-technical and easily understood.

The SI will avoid coercion, will not appear to waive the subject’s legal rights in any way, will allow enough time for the subject to inquire about the details of the clinical study, ask any questions and make the decision to participate or not in the clinical study.

Should the subject decide to participate in the clinical study, the informed consent form will be signed and personally dated by the subject and the researcher who conducted the informed consent discussion. A copy will be given to the subject. Any significant relevant new information that arises during the clinical study will be provided to the subject and their consent to continue will be sought.

# Statistics

A separate detailed statistical analysis plan will be developed in cooperation with the study statisticians and will be published after approval from the steering committee, no later than before the first scheduled interim analysis.

A group sequential design with adaptive sample size modification is chosen to allow for the possibility of stopping the trial early due to significant differences in the primary endpoint or 30-day survival rate between the groups and to re-estimate sample size at the interim analyses to maintain the desired statistical power. This is described in chapter 9.3. Adaptive design has been advocated and used in clinical research due to several reasons ^40–43^. This includes cost-effectiveness regarding time and money, it might require fewer participants, ethical argument due to safety and efficacy of the trial intervention and that it might mimic the real-life medical practice more than a traditional randomized control trial.

## Statistical hypothesis

Null hypothesis H_0_: There is no difference in rate of return of spontaneous circulation between the control group (advanced cardiovascular life support only) and the intervention group (advanced cardiovascular life support + REBOA).

Alternative hypothesis H_A_: There is a difference in rate of return of spontaneous circulation between the control group (advanced cardiovascular life support only) and the intervention group (advanced cardiovascular life support + REBOA).

## Sample size

One prospective pilot feasibility study report that the procedure is feasible in the pre-hospital setting ^1^. This pilot study was without a control group, the number of patients was only 10, with 60 % rate of ROSC and with one survivor after 30 days. No other prospective studies are currently known to us. Therefore, we lack prospective data to allow a reasonable estimation of sample size for a conclusive trial design.

In Norway, the current overall rate of ROSC is 32% ^4^. Patient’s eligible for a REBOA procedure is however a cohort of this data material, which is defined by the inclusion and exclusion criteria, the duration of the dispatch and necessary procedure duration. The cohort is described in detail in a needs assessment for REBOA in non-traumatic OHCA in Norway and the reported rate of ROSC is markedly reduced to 18% ^33^.

The sample size needed to demonstrate a clinically relevant doubling of ROSC from 18% to 36%, with 0.80 power and a significance level of 0.05, is 94 patients in each group ^44^ (Figure 2).


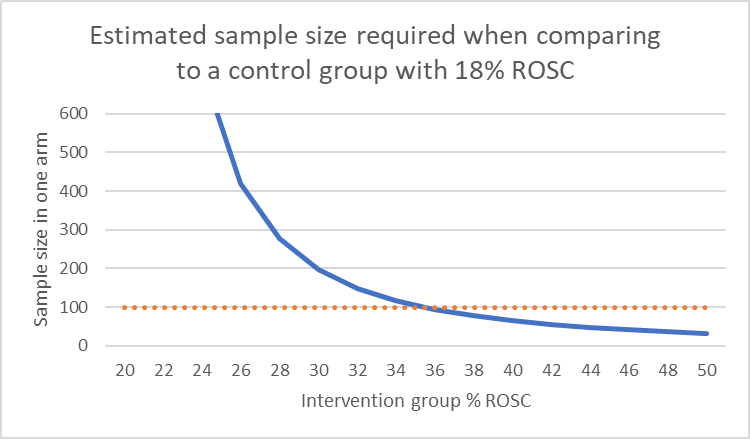


*Figure 2. Estimated sample size with return of spontaneous circulation as primary endpoint. Horizontal line is drawn at the selected sample size n=100.*

We therefore choose to include 100 patients in each group.

## Interim analyses

Three interim analyses will be performed, after 30, 60 and 90 patients in both the intervention and the control group is included. No specific time schedule for interim analyses is set. The important variables to assess is the primary endpoint and 30-day survival with good neurologic outcome.

We will consider recommending the sponsor to stop the trial after interim analysis if there is a difference in primary endpoint or 30-day survival between the groups, with a significance level following the O´Brien-Fleming approach ^45,46^.

From the second interim analysis, we will perform a sample size calculation, based on the assumption that the current difference in primary endpoint between the two groups will persist. If the sample size needed to prove a difference is more than three times the planned sample size (with 0.80 power and a significance level of 0.05), we would consider recommending the sponsor to stop the trial due to futility.

On the last interim analysis, we will re-estimate the sample size in case of a non-significant difference on the primary endpoint. If the estimated sample size needed to prove a difference between the groups is above 100, but equal to or less than 150 in each group (with 0.80 power and a significance level of 0.05), the final sample size will be modified and we would recommend the sponsor to continue the trial until the modified sample size is reached.

## Measures to minimize bias

**Selection bias**

This is a randomised study and randomisation happens on scene. This is important to minimise selection bias. Randomisation procedure is described in chapter 10.1.

**Performance bias**

The planned post-ROSC treatment, and treatment after admission to hospital, will not differ in the two groups. This is important to minimize performance bias. However, the intervention group will have the REBOA equipment in place when admitted and this will warrant specialized care and removal. It is also possible that the introducer sheath will be used for quick access to PCI or ECMO. We will gather in-hospital data to assess these eventual differences in in-hospital treatment.

**Exclusion bias**

The information regarding informed consent, follow-up of patient survival and other patient data, interview with performing personnel and procedure data is performed by a small number of personnel. Only one or two persons on each study site is responsible to collect this data. This same person is responsible for the registration of data to the CRF. This contributes to minimise exclusion bias.

**Detection bias**

The randomisation is blinded from the statisticians that perform the analyses. This contributes to reduce detection bias.

## Statistical design

The primary analysis will be according to intention-to-treat (ITT) principle, to compare the outcome between all participants randomised to the control group and the intervention (REBOA) group, i.e. all patients in the REBOA group will be included in the analysis regardless of actual occlusion of aorta. Per-protocol analyses will be considered if a considerable amount in the REBOA group has deviated from protocol and hence may undermine the validity of the ITT analysis. This deviation from protocol could result from that the patient either achieved ROSC or was declared dead before aortic occlusion, or the procedure was aborted of any reason.

The primary endpoint and other dichotomous secondary endpoints will be analysed using hypothesis testing. Continuous variables (end tidal CO2 values and blood pressure) will be analysed by regression methods.

## The level of significance and power of the clinical study

It is not known if the REBOA can only be of benefit, it is also possible that it may decrease the chance of good outcome. Therefore, a two-sided test with significance level of 0.05 is chosen in the analyses. On the interim analysis, a significance level following the O´Brien-Fleming approach is chosen ^45,46^.

## Expected drop out rates

The randomisation and intervention are performed before informed consent can be obtained. Due to earlier experience with studies in our centre involving cardiac arrest patients and deferred consent ^47^, and the experience from the pilot trial, we expect a very low drop-out rate. The estimate is therefore set to 5%.

## Specification of exploratory subgroups

This is the first large dataset to be collected on this intervention. Any subgroups that may have enhanced or reduced effect of the intervention is currently unknown. To assess the possible modifiers of the intervention effect, the following exploratory analyses will be used:

- Age
- Sex
- Type of initial rhythm (VF/VT or PEA/AS)
- Time from debut of arrest to aortic occlusion
- Study site
- Type of catheter used
- Hypoxemia as probable cause of arrest

These will be analysed using generalised linear models. This includes multiple regression analyses, which enable multiple imputation if necessary.

## Treatment of missing data

Missing data will be reported in the primary publication.

There will be no missing data on the primary endpoint, due to the design of the case report form.

Multiple imputation will be considered if substantial amount of data is missing.

## Min/max number of subjects per centre

No minimum or maximum number of subjects per centre is set.

# Project methodology and overall design

The purpose of the study is to assess the efficacy of REBOA as an adjunct treatment in non-traumatic cardiac arrest and the effects of this intervention on ROSC. This is not a trial to test any medical device, nor to compare the effect of different REBOA catheters.

This is a prospective, randomised, parallel group, multi-centre, phase II clinical trial. Patients are randomised in a 1:1 ratio to be included to the control group or the intervention group (Figure 3). The control group receives ACLS according to national guidelines. The intervention group receives ACLS according to national guidelines and the REBOA procedure as an adjunct treatment.


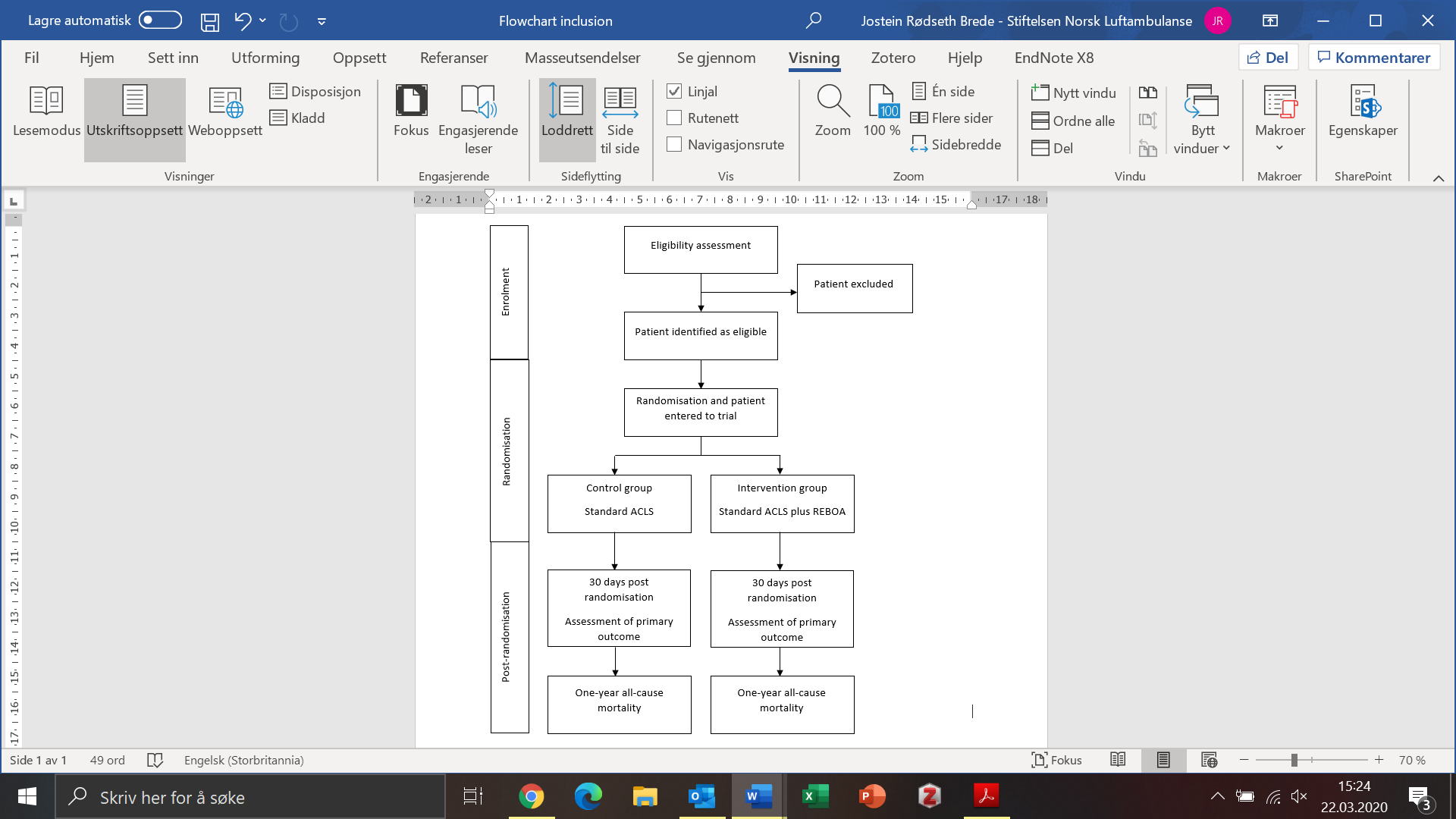


*Figure 3. Flow-chart of patient allocation*

Because of the nature of the intervention, it is impossible to blind the performer of the REBOA procedure. Further is it not practically possible to blind the patient for treatment group. Statisticians that perform the analysis of primary and secondary endpoints will be blinded.

The research project is estimated to last for 10 years. This includes start-up and education of the study centres, enrolment of patients, evaluation of last included patient one year after randomisation and five years retention period of study documentation.

## Randomisation procedure

Patients will be allocated in a 1:1 ratio between the two study groups. A permuted block randomisation method stratified by site will be used to allocate eligible patients to either the control group (treatment 1) or the intervention group (treatment 2). This will be as sealed envelopes to be selected on scene if the patient is eligible for randomisation. Each site will have envelopes in each appropriate intervention kit (one in each either helicopter, rapid response car or both). Randomisation will be performed by KlinForsk, Clinical Research Unit, Central Norway. The random allocation sequence is generated using a Microsoft Access data base by KlinForsk. No investigator has access to the allocation sequence.

The envelopes will not be able to see through or open without breaking the seal. The envelopes will be collected by members of the project management and distributed to the site investigator on each study site for placement in the REBOA study kit. An accountability form for all envelopes will be in place. Unopened envelopes will be returned to KlinForsk.

## Medical devices in the study

In advanced cardiovascular life support several medical devices are used for treatment and diagnosis. All these devices will be used on patients in both groups of this trial. Product specifications and exact producers/ product numbers/ batch codes of devices other than REBOA catheters will not be recorded. The sponsor of this trial takes no responsibilities in the funding, provision, approval or other aspects regarding devices other than the REBOA catheters in this trial.

### Medical devices in both groups

- Mechanical chest compression systems
- Monitoring equipment
- Cerebral oximetry equipment
- Defibrillators
- Ultrasound
- Central and peripheral venous catheters
- Arterial catheters
- Intraosseous needles
- Endotracheal tubes
- Supraglottic airways
- Bag and mask for ventilation
- Others

### Medical devices in the intervention group only

In addition to equipment used in both groups, a REBOA balloon catheter and procedure kit will be used. Several balloon catheters are available. Currently, two catheter types are considered for use in this trial:

- the REBOA Medical TM 20 mm balloon, REBOA Medical AS
- the ER-REBOA Catheter, Prytime Medical

Product information and instructions for use are attached (appendix 29.3).

Other catheters, e.g. the Tokai Occlusion Balloon Catheter or any new catheters from different producers that may be available during the trial period, will be considered for use. All catheters will be used according to the CE/FDA approval.

It is an obvious advantage that the same equipment is used both pre-hospital and in-hospital at a study site. Local variations in in-hospital use can therefore dictate a change in catheter type at a study site. This will be decided by agreement of the hospital in question, the site investigator and the sponsor.

### Kit for REBOA intervention

The necessary equipment to perform the REBOA procedure is gathered in a procedure kit. This is as supplied by the manufacturer (Prytime or Reboa Medical) and is not altered. The kits are identical on all study sites using the Prytime or Reboa Medical catheter, respectively.

The study equipment (catheters and REBOA kit) will be clearly labelled “for REBOARREST trial purposes only”, see label in appendix, chapter 29.4.

A separate system for accounting of all catheters will be in place at each study site. The site investigator or designee (SI) has the responsibility for proper handling and storing of the REBOA equipment at each study site. This includes follow-up that enough equipment is in stock. The REBOA kit contains all necessary equipment to perform the procedure. When needed, the SI contacts the project management to order supplies of equipment.

Other necessary equipment (chapter 10.2.1) is considered standard equipment handled at the study sites according to their normal routine. The study equipment will only be used to perform the procedure in subjects included in the clinical study. If the equipment is used for other indications at a study site, the sponsor takes no responsibility for the performance of the equipment or the patient’s outcome.

## Expected duration

The study trial period for each patient is one year after randomisation. After 30 days, the survival rate and adverse reactions during this 30-day period will be reported. After one year a chart review will be performed to assess all-cause mortality.

The trial is expected to open first site winter 2020/2021. Inclusion of patients is estimated to take three years. Data registration, patient follow up one year after last inclusion and five-year data retention period necessitate a total study period of 10 year.

# Study procedures

Treatment 1 – the control group

The control group will receive treatment as described in the guidelines from the European Resuscitation Council^10^, the Norwegian Resuscitation Council^9^, and other local national guidelines.

Treatment 2 – the intervention group

The intervention group will receive the same treatment as the control group. In adjunct, this group will receive the intervention resuscitative endovascular balloon occlusion of the aorta (REBOA).

The insertion of the CE/FDA-approved catheter will follow the procedure as described from the producer. Other catheter that may be used during the trial period will be inserted according to the procedure described from the producers. The instructions for use for each of the currently used catheters are attached (appendix 29.3).

An outline of the REBOA procedure is described in section 11.1.2.

## By Visit

### Before Treatment Starts

**Education and training of personnel**

All involved personnel are regularly part of resuscitation teams both in real life and in training scenarios. This trial will therefore not include any mandatory additional training in standard ACLS.

The pilot study ^1^ demonstrate that the REBOA procedure is feasible in the patient cohort. The P-EMS crew had participated in a structured educational program ^48^. A similar educational program will be held in all study sites and all performers (physicians and paramedics) must complete and pass this program before they can perform the procedure in study subjects. A certificate of approval will be handed to all personnel that complete the program.

All study sites will be equipped with the necessary cannulation equipment and a training dummy to facilitate regularly procedure training during the trial period.

### During Treatment

**Outline of a REBOA procedure at an out-of-hospital cardiac arrest**

The ambulance personnel and the P-EMS crew establish ACLS as per national guideline at site. Either manual compressions or a mechanical chest compression is used. Airway management is handled by either endotracheal intubation or a supraglottic airway device. Intravenous or intraosseous access on the upper body is established. Cerebral oximetry is used, if available. If the patient is eligible for inclusion, the patient is randomised by that the physician opens the next numbered sealed envelope from the REBOA equipment. The randomisation procedure is described in section 10.1.

The paramedic prepares the REBOA-equipment, while the physician prepares the ultrasound machine and examines the patient for visible femoral vessels. Most physicians are right-handed and therefore will prefer to cannulate the patient’s right femoral artery, while placed on the patient’s right side. The paramedic prepares the equipment at the patient’s foot. The femoral inguinal area is washed with antiseptic and a sterile drape covers the patient, from abdomen to distal.

Arterial cannulation is performed ultrasound guided, with out-of-plane technique (Figure 4).


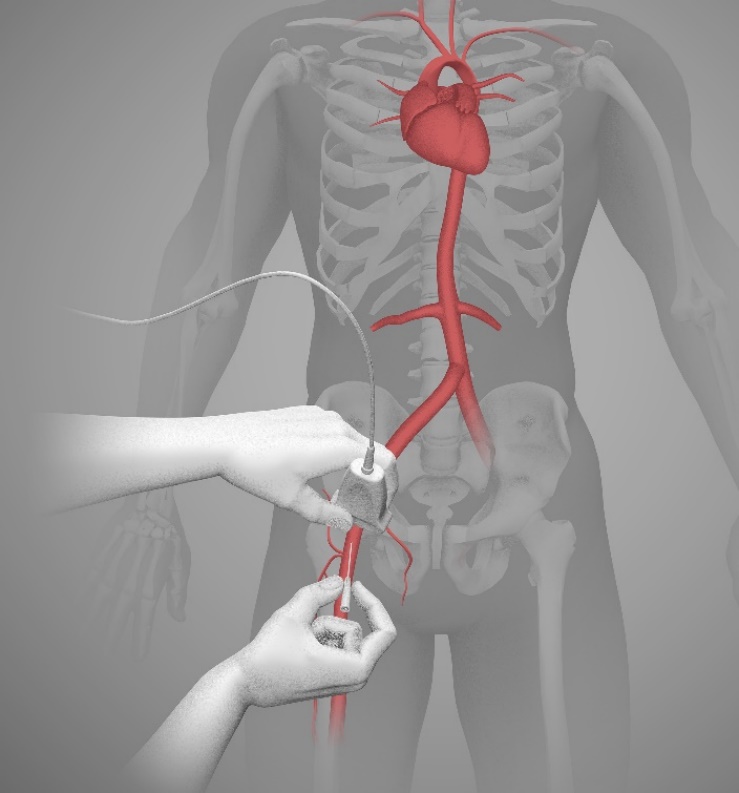


*Figure 4. Ultrasound guided femoral arterial cannulation*

After the femoral artery and vein is identified, the artery is cannulated with a 19 G needle and a flexible guidewire is inserted 60 cm. The cannulation can be performed in the ventilation phase of CPR or a 10-20 second pause in chest compression is allowed for. This hands-off time is considered common practice. After the guidewire is placed, the needle is removed, and ultrasound images of the guidewire position is obtained and stored. A scalpel is used to make a small incision next to the guidewire, to widen the entrance point. A 7 Fr introducer sheath is inserted over the guidewire and the stylet is removed (Figure 5).


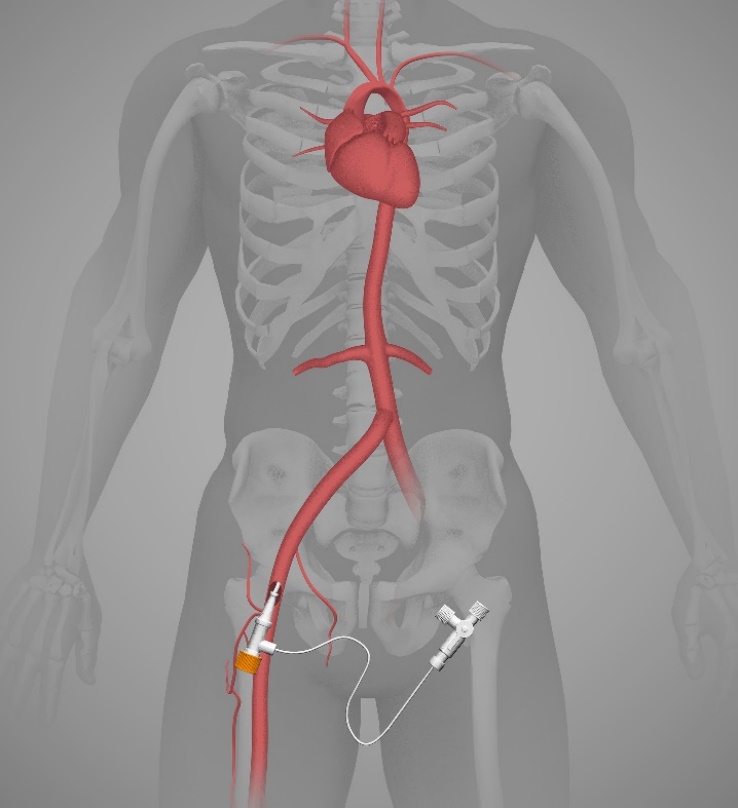


*Figure 5. Insertion of introducer in femoral artery*

If the catheter in use is the ER-REBOA catheter, the guidewire is also removed, and the balloon sheath is placed. If the catheter is the REBOA Medical balloon, the guidewire will remain and the catheter is placed over the guidewire, through the introducer. The balloon sheath is inserted 50 cm for a Zone 1 aortic occlusion (Figure 6) ^49^, by use of measure marks on the catheter. Zone 1 is favoured for best haemodynamic effect ^49,50^.


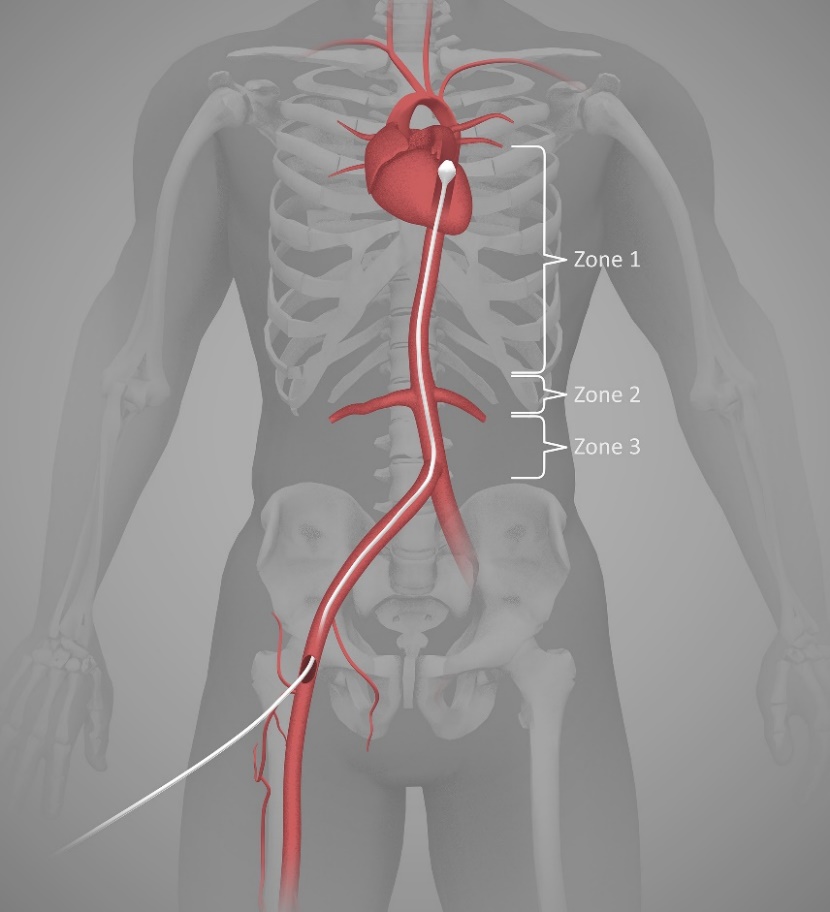


*Figure 6. Zone 1 aortic occlusion*

If applicable, arterial pressure measurements from the distal tip is obtained. Pressure transducer is prepared as per standard routine. Before the balloon is inflated, a palpable pulse check in left radial artery is performed. The balloon is then inflated with 0.9% NaCl or until resistance is felt (max 15 ml if REBOA Medical balloon and max 8 ml if ER-REBOA balloon). The radial pulse check is again performed. If a palpable pulse is present, the location of the balloon is accepted. If a present pulse disappears after balloon inflation, the balloon is deflated, the balloon sheath is withdrawn 5 cm and inflation/pulse check is performed. When in place, the introducer is sutured to the skin and the balloon sheath is attached to the patient’s leg/clothes using tape, clamp or other available equipment.

Invasive blood pressure is measured continuously before and after balloon inflation, when applicable.

If ROSC is achieved, the balloon is slowly deflated, over 30 seconds. Post-ROSC treatment is then performed as per standard routine.

The decision to discontinue resuscitation efforts are performed as per national guideline at site, regardless of the patient’s inclusion in this trial.

The physician must complete a checklist (appendix 29.5) before leaving the scene. This includes these items: Inclusion/exclusion criteria, Video of guidewire placement stored (if applicable), Arterial pressure curves registered (if applicable), EtCO2 measurements noted and ROSC (yes/no).

### End of Study Visit

After admission to hospital or when pre-hospital care has ended, the physician will notify the site investigator or designee (SI) of any included patient. The SI will then take over responsibility for data collection.

The SI will debrief the physician at an appropriate time and will provide study information to the next of kin and access deferred consent, as described in chapter 8. The SI will gather and register necessary data in the case report form (CRF) and upload the ultrasound file to the secure data storage.

If adverse events occur, the SI will follow as per protocol.

## After End of Treatment (Follow-up)

We use a 30-day follow-up period and any adverse effects related to the procedure is likely to be found during this period. The follow-up is performed by the site investigator or designee. The SI will perform a chart review and a telephone interview, or face to face meeting if applicable, with the patient or the patients’ legal representative.

The SI will also perform the follow-up one year after randomisation and register the result in the CRF.

### Follow-up medical care

All patients will receive standard clinical care after randomisation or after the intervention is performed.

The patients included in the intervention group will eventually need removal of the REBOA introducer and catheter. In Norway this is performed by interventional cardiologist or interventional radiologist, depending on intra-hospital policies. This will be agreed upon with the project management, the site investigator and the relevant Head of Clinic before the study site can enter the study. Foreign study sites will provide the project management with documentation on how this will be handled before the study site can enter the study.

## Trial Discontinuation

The whole trial may be discontinued at the discretion of the sponsor in the event of any of the following:

- Occurrence of adverse events unknown to date in respect of their nature, severity and duration
- Medical or ethical reasons affecting the continued performance of the trial
- Difficulties in the recruitment of patients
- Changes in funding

If the interim analysis report significant group difference in primary endpoint, the study can be stopped. A recommendation to the sponsor to stop the study will only happen after a face to face meeting of the project management.

The study may be suspended or terminated by a site investigator at any time. The SI can only suspend or terminate the study at his/her study site.

The study may be suspended or terminated if serious safety issues are raised. This includes malfunction of equipment, indications of misuse of equipment or inappropriate performance skills in one or more of the physicians.

The data monitoring committee can recommend the sponsor to suspend or terminate the study if they find grave and unsolvable safety issues.

## Laboratory Tests

Several blood samples are commonly drawn from patients admitted after OHCA. We will register results from these samples, both to address possible differences between the groups, but also to assess a potential visceral hypoperfusion in the intervention group, as discussed in chapter 13.

There will be no biobank established and all laboratory test in this trial will be performed in accordance with laboratory standard procedures.

## Concomitant therapy

Concomitant therapy and medical interventions in the pre-hospital phase will be registered in the CRF.

No medication excludes participants from being included in this trial. Medications used by patients prior to inclusion in this trial, such as anticoagulants, will be recorded if information is available at the time of inclusion. All medications administered as part of the cardiopulmonary resuscitation is recorded in the CRF. Medications will be recorded as generic name, dose and route of administration.

Cardiopulmonary resuscitation may include both medications and techniques like thoracotomy or dual sequential defibrillation. These treatments will not differ in the two study groups. Concomitant therapy and medication between admission to hospital and evaluation after 30 days will not be registered.

# Data collection and processing

## Data handling

**Case Report Forms (CRFs)**

Data from each patient will be entered into an electronic database, a web-based case report form (webCRF3, <https://webcrf3.medisin.ntnu.no/>). This is developed and handled by the department KlinForsk, Clinical Research Unit, Central Norway. KlinForsk is authorised as data processors by the Norwegian Centre for Research Data. KlinForsk will set up necessary accounts to permit access to the CRF.

**Source Data**

Data are collected from

- Air ambulance journal the physician submits after an out-of-hospital cardiac arrest
- Ambulance journal
- Utstein pre-hospital cardiac arrest journal
- Data file from patient monitor used (in Norway this is most often Tempus Pro^TM^, RDT, United Kingdom or Corpuls3, GS, Germany). The monitor used may differ between study sites.
- Data file from cerebral oximetry system, e.g. ForeSight, Edwards Lifesciences Corp., USA. The system used may vary during the study.
- Checklist with registrations and inclusion criteria for the procedure (appendix 29.5).
- The site investigator will debrief each physician after the procedure, following a semi-structured interview template (appendix 29.6).
- The ultrasound images from the ultrasound machine used.
- The electronic patient journal, including laboratory tests.

**Storage of Study Documentation**

All data handling will follow strict security and privacy controls in compliance with Norwegian health and privacy laws. Electronic data gathered specifically for this trial (ultrasound images, copies of the monitor files) will be uploaded and stored in a private REBOARREST data space in HUNT Cloud, a part of HUNT data centre (<https://www.ntnu.edu/mh/huntcloud/about>). Access will be restricted to authorized users only on a need to know and need to do basis. Data will be protected in compliance with HUNT Cloud's ISO certificates for security and quality management (ISO 27001 and ISO 9001) as of today applicable to 161 security controls for which two-factor authentication is one.

Hard copies of patient documents, paper CRF’s or other printed document that may include identifiable information will be stored at a locked location at each study site or at the study coordinating centre, Rosten, Trondheim. Only the site investigator has access to this information. The Project Manager will have access to the information stored at the study coordinating centre.

Original trial documents (signed consent forms, notes from interviews with performing physicians, copies of patient journals, records of serious adverse events or other relevant correspondence) will be stored at the study sites or at the main research centre at Rosten, Trondheim, for five years to allow for inspection by relevant authorities.

When the data, either paper or electronic, are archived, the site investigators will maintain confidentiality of the data and prevent accidental destruction of documents. Paper and electronic data will be retained at least five years after the trial is finished.

## Data collection during study period

Data will be registered according to this schedule.


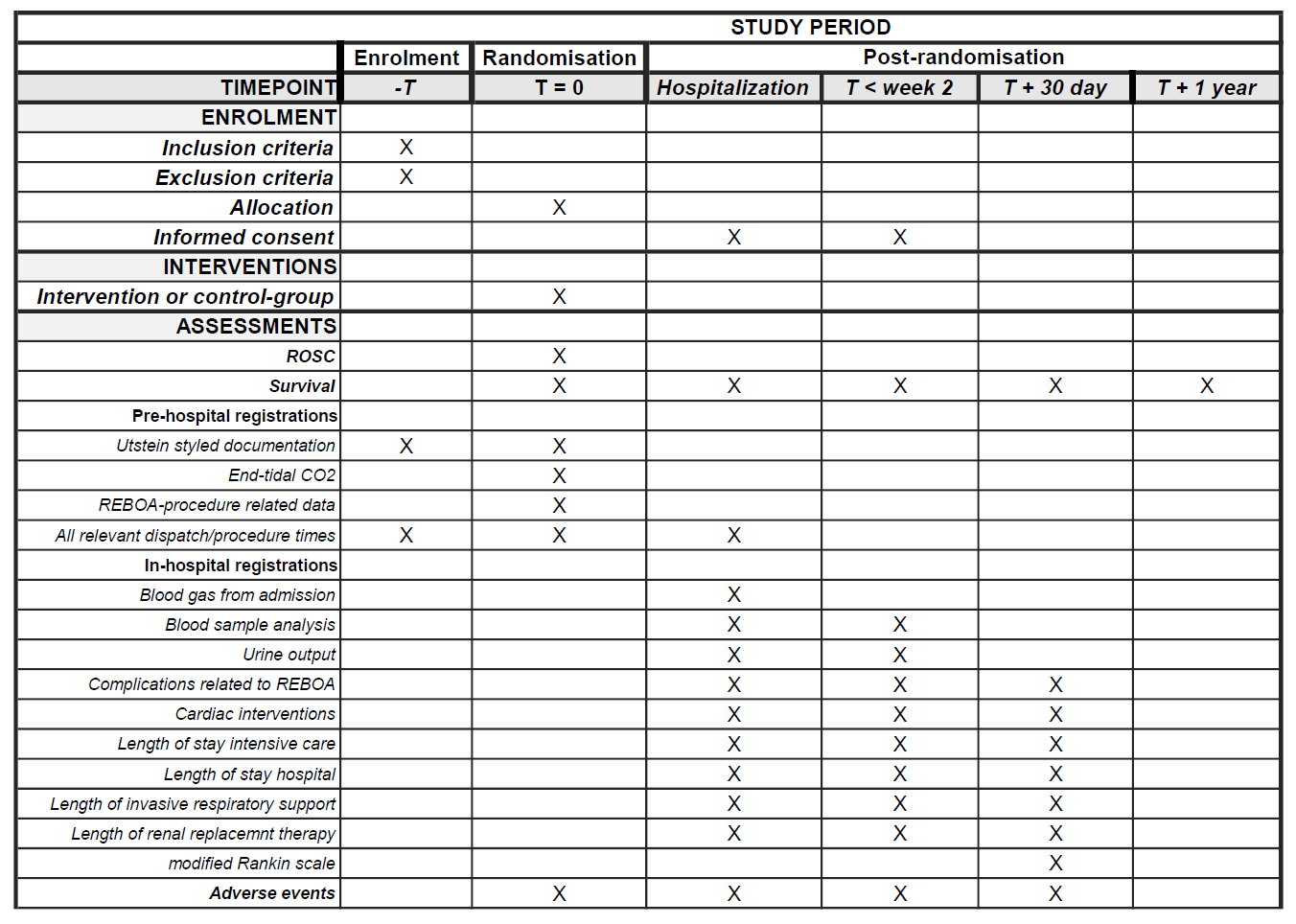


These data points will be registered:

| **All patients** - data reported after randomisation | |
| --- | --- |
| Age |  |
| Gender |  |
| Habitus | *Cachectic, Normal, Adipose* |
| Location of the incident | *Public outdoors, public indoors, private indoors* |
| All concomitant medication and dose used |  |
| Route of administration | *Intravenous, intraosseous* |
| All relevant times | *DD.MM.YYYY: hh:mm* |
| Time of dispatch |  |
| Time of arrival |  |
| Time of randomisation |  |
| Cardiac rhythm at randomisation and fluctuations in rhythm during resuscitation | *VF, VT, PEA, AS* |
| Suspected aetiology of arrest | *cardiac, respiratory, intoxication/poisoning, non-traumatic haemorrhage* |
| Number of defibrillations | *n* |
| Compression machine used | *yes/no* |
| Airway management (main method) | *Supraglottic airway, Endotracheal intubation* |
| ROSC more than 20 minutes | *yes/no* |
| Data file from monitor used |  |

| **Control group - additional data** | |
| --- | --- |
| End-tidal CO2 measures after randomisation and after ROSC | *kPa* |
| Other relevant times | *DD.MM.YYYY hh:mm* |
| Time of ROSC |  |
| Time of departure from scene |  |
| Time of admission to hospital |  |

| **REBOA group - additional data** | |
| --- | --- |
| Space to perform procedure | *Good/acceptable, Challenging, Impossible* |
| Visibility/light conditions | *Good/acceptable, Challenging, Impossible* |
| Temperature | *Good/acceptable, Challenging, Impossible* |
| Wind/rain conditions | *Good/acceptable, Challenging, Impossible* |
| Available resources on the scene that were used | *Bystanders, Another doctor, Ambulance, Fire brigade* |
| Ultrasound image obtaining | *Good/acceptable, Challenging, Impossible* |
| Data file from ultrasound machine |  |
| Cannulation site | *Left, Right* |
| Numbers of cannulation attempts | *n* |
| End-tidal measures | *kPa* |
| Before and after inflation of balloon, and after ROSC |  |
| Palpable pulse in left radial artery | *yes/no* |
| before balloon inflation |  |
| after balloon inflation |  |
| Other relevant times | *DD.MM.YYYY hh:mm* |
| Time of balloon occlusion |  |
| Time of ROSC and deflation of the balloon |  |
| Time of departure from scene |  |
| Time of admission to hospital |  |

| **Delivered to hospital - additional data** | |
| --- | --- |
| Blood gas from admission |  |
| pH |  |
| pCO2 | *kPa* |
| pO2 | *kPa* |
| SaO2 | *%* |
| Base Excess | *mmol/L* |
| HCO3 | *mmol/L* |
| s-Lactate | *mmol/L* |
| s-K | *mmol/L* |
| s-Na | *mmol/L* |
| s-Ca | *mmol/L* |
| Glucose | *mmol/L* |
| Blood sample analysis, up to 30 days, with time and date for samples | *DD.MM.YYY hh:mm* |
| White blood cells | *x10^3^/uL* |
| Blood platelets | *x10^3^/uL* |
| CRP | *mg/L* |
| Neuron Specific Enolase (NSE) after 48-72 h | *ug/L* |
| Troponins | *ng/L* |
| CK-MB and total-CK | *ug/L and U/L* |
| PT-INR |  |
| ASAT, ALAT | *U/L* |
| Bilirubin | *umol/L* |
| Albumin | *g/L* |
| Creatinine | *mmol/L* |
| Estimated GFR |  |
| Urine output, up to 30 days | *ml/day* |
| Complications related to REBOA equipment, with time and date for each complication | *yes/no, DD.MM.YYYY hh:mm* |
| Local haemorrhage requiring treatment |  |
| Lower limb ischemia |  |
| Infection requiring antibiotic treatment |  |
| Aortic dissection, rupture or injury |  |
| Femoral vessel dissection, rupture or injury |  |
| Surgical intervention/repair needed |  |
| Need for amputation  Other |  |
| Cardiac interventions  REBOA equipment used for in-hospital procedure | *PCI, IABP, IMPELLA, ECMO, other*  *Yes/no for each procedure* |
| Length of stay at intensive care unit during the 30-day period after randomisation | *number of days* |
| Length of stay in hospital during the 30-day period after randomisation | *number of days* |
| Length of invasive respiratory support during the 30-day period after randomisation | *number of days* |
| Length of renal replacement therapy dependency during the 30-day period after randomisation | *number of days* |
| Modified Rankin scale at day 30 | *0 to 6* |
| Survival one year after randomisation | *yes/no* |
| Results from radiologic exams first 30 days |  |
| Results from echocardiography first 30 days |  |
| Data from autopsy, if performed |  |

## Procedures for data review and editing

After randomisation each patient will be given a unique study number. The site investigator or designee will handle an enrolment log (*Subject ID log* and *Screening log*, appendix 29.7 and 29.8) which include the patients name, date of birth and study number. Subsequent data will be identified only by the study number. No identifiable data will be entered to the CRF. The enrolment log will be stored separately at each study site.

Data that are not uploaded to the CRF will be handled by the site investigator only. The SI is responsible for an interview of the involved personnel, to gather the necessary data and to edit and upload this to the secure database and entry into the CRF. The data file from the monitor used in all randomised patients, and the ultrasound images from the patients in the intervention group, are preserved and protected in the REBOARREST data space in HUNT Cloud. Data integrity policies and data integrity controls are to ensure that investigators cannot remove raw data, and that duplicate copies can be made available for accountability audits.

If an investigator for some reason must withdraw from the study, his/her responsibility is transferred from this investigator to the site investigator and the project management, which will make efforts to appoint a new investigator, if necessary.

## Specified retention period

Electronic database and study archive are maintained for at least five years. Individual patient data records will be handled according to the legislation of each participating country.

# Safety assessment

## Risks and benefit

This is an invasive and demanding intervention in a critical ill population and risk will apply. The project management has conducted an analysis of the benefit and risks of the study.

### Specific risk for interruption of ACLS quality

It is imperative that a new intervention should not interact negatively on the performance of the resuscitation. The REBOA procedure must therefore not influence negatively on the quality of the ACLS, all patients must receive standard pre-hospital care. The procedure may interrupt the ACLS because focus is drawn from the resuscitation to the REBOA procedure performed.

A structured educational program has been designed and formally evaluated ^48^ and all physicians that will include patients will be tested and certified before they can perform any clinical REBOA procedure. This ensure that all participants receive a standardized level of education and training, regardless of study site.

The risk of interruption of ACLS quality is most importantly mitigated through the structured educational program, regularly ACLS and REBOA training, simulation exercises and information to the emergency services at each study site. The pilot study demonstrates that REBOA is feasible in the pre-hospital setting, and that the intervention did not influence the quality of the ACLS ^1^. In the pilot study a three-step safety program was conducted.

In this trial, a similar safety program will also be conducted:

- After each randomised patient, the performing physician will be interviewed by the SI, following a semi-structured interview (appendix 29.6). Any safety concerns from the performing physician will be addressed.
- The ultrasound images obtained from patients in the intervention group will be reviewed by the SI and members of the project management or steering committee to ensure arterial placement of the guidewire.
- A case review of each randomised patient using all available information by an external expert panel consisting of an interventional radiologist, an intensivist and a pre-hospital physician. Hands-off time and duration of CPR effort will be specifically assessed.

### Specific risk of venous placement of REBOA catheter

One other anticipated risk is erroneous venous placement of the REBOA catheter. The femoral artery lies adjacent to the femoral vein. Femoral vein cannulation and subsequent occlusion of the vena cava inferior result in reduction of cardiac preload and must be avoided. Ultrasound guided cannulation is therefore mandatory. It is also mandatory to have two visible vessels before cannulation. With only one visible femoral vessel, it is impossible to establish if the vessel is a vein or artery. Out-of-plane cannulation technique is also mandatory, as this provides view of two vessels simultaneously. An in-line cannulation technique will not ensure arterial cannulation.

Any eventual venous cannulation can easily be used as a central venous line. If the balloon catheter is erroneously inflated in the vena cava inferior, we expect a rapid decrease in EtCO2.

The Norwegian national P-EMS include anaesthesiologists ^51^. These physicians will regularly be part of the resuscitation team at OHCA and provides a pre-hospital competency in establishing central vascular lines ^52^. Foreign study sites may involve physicians from other specialities than anaesthesiology, e.g. emergency physicians. However, all physicians involved in this study is familiar and competent in the Seldinger technique. Femoral access is preferable during CPR because resuscitation efforts limit access to the upper thorax and neck. Direct visualization of the femoral vessels with ultrasound facilitates catheterization during CPR ^53^. A blind, landmark-oriented approach to femoral catheterization is difficult in patients with low blood pressure or no palpable femoral pulse ^54^ and risks a higher rate of complications because femoral pulse is not located in 40% of CPR patients ^53^. A review-article reports that ultrasound guidance for femoral artery catheterization was associated with 49% reduction in overall complications, including hematoma and accidental venous puncture. It was also associated with 42% improvement in the likelihood of first-attempt success ^55^. Ultrasound is therefore considered mandatory in arterial cannulation during CPR.

### Other risks

This study adds a new treatment to patients with critical illness, in a setting where informed consent by the patient is impossible. It is imperative that no patients are subject to greater risks than the ones already related to the critical illness. Insertion of similar intravascular catheters are already in use for other indications, e.g. invasive arterial blood pressure measurements, and will in this study be performed by physicians well experienced in establishing vascular access in critical ill patients. The patients are at risk for local complications such as bleeding at the femoral site, but these complications, shared by other interventional procedures such as percutaneous coronary intervention (PCI), are not expected to represent serious adverse events.

Risk of lower limb ischemia

The REBOA technique is not new. The equipment is well used, in a range of indications and patient ages. Previously reported complications were after procedures where large gauge equipment was used, 10 to 14 French ^49,56^. It has been reported that these large sheaths may be associated with severe complications, including lower extremity ischemia and amputations ^57^. These complications may be related to the near-occlusive diameter of these larger sheaths, the length of time they remain in the artery, the location of insertion, and potential damage that can be caused during insertion. Studies show that smaller sheaths, 7Fr, are both safe and effective. These sheaths can remain in place for a prolonged time after insertion without severe complications ^58,59^. One study showed no vascular complications using 7Fr sheaths ^60^. A Norwegian study show that short introducer length (11 cm) was strongly associated with complications (local thrombus formation in the iliac or femoral artery) ^61^. One episode is reported of a tear of the aorta wall due to oversized balloon ^59^. The descending aorta is approximately 25 mm in width in the age span of the patient eligible for inclusion ^62,63^. The balloon catheter will reach no more than 10-20 cm outside the semi-rigid introducer, and both introducer and catheter will be fixated after insertion. Migration of the catheter with a theoretically potential damage to the aorta is therefore not likely. To minimize the risk of complications such as aortic rupture or local complications, a 7Fr introducer with a 20 mm balloon will be used.

Risk of organ ischemia

Aortic occlusion will limit blood flow to organs distal to the occlusion. One study advocated the use of repetitive deflations of the balloon every 10-15 minutes to minimize the risk of ischemic complications ^61^. We will not apply this regime, due to the low output state and marginal cardiac output generated with external chest compressions. The occlusion time will be limited, as clinical decisions for other resuscitation procedures, whether to stay on scene or continue CPR during transport to hospital, or when to terminate CPR efforts, is performed as in routine care, regardless of treatment with REBOA or not. One patient in the pilot study experienced ischemic colon and a hemicolectomy was performed ^1^. The incident was reported to the proper health investigational authorities. The following investigation concluded that the REBOA procedure was not the cause for this ischemic colon.

Risk of occlusion of carotid artery

The left subclavian artery springs from the aorta distal to the left carotid artery. A pulse check in left radial artery is performed before and after occlusion. If radial pulse is present after occlusion of the aorta, it is not likely that the balloon occludes carotid circulation.

Risk of increased bleeding proximal to balloon occlusion

One case report describe an intracranial haemorrhage in a multi-trauma patient receiving REBOA in the emergency department ^64^. REBOA could worsen bleedings proximal to the occlusion and we will not include patients where a neurologic injury (e.g. cerebral haemorrhage) is believed to be the aetiology of the cardiac arrest.

Risk of infection

The physicians are skilled in pre-hospital invasive techniques done under sterile condition (e.g. central vein punctures, pleura drainage catheters), and the risk of severe blood infection due to the procedure is considered low.

Risk to study personnel

The physicians are skilled in using equipment such as scalpel and needles. They will all participate in an educational program to ensure a safe and efficient REBOA technique. On each study site, training phantoms for REBOA technique training will be installed. This facilitates regularly team training on the procedure. Risk for mechanical injuries on the P-EMS crew or unnecessary injuries on the patient is considered low. If any needle puncture injuries should occur on the personnel, the procedure for such events at the local hospital will be followed.

### Autopsy

As in all pre-hospital managements of cardiac arrest, it is likely that several patients will be pronounced dead on scene or after ACLS efforts, regardless of REBOA placement. An autopsy could potentially provide information both on cause of death or complications of the ACLS or REBOA procedure. In Norway, autopsies are regulated in the “Autopsy Act” ^65^. This act separates “forensic autopsy” and “medical autopsy”. A forensic autopsy might be indicated if the body is of unknown identification or suspected criminal behaviour prior to the cardiac arrest. The decision to perform a forensic autopsy is made by the local police. Most pre-hospital deaths will not fall into this category. A medical autopsy is indicated for scientific reasons or for research. However, information to and consent from the next of kind is needed to perform a medical autopsy. It is not likely possible for the P-EMS crew to assess this information and consent in the pre-hospital setting. Also, it might not be ethically justified to use a pre-hospital emergency resource (ambulance or P-EMS crew) to transport the deceased to a university hospital to perform an autopsy.

However, this is a novel use of an invasive intervention and any information of potential harm caused by the REBOA equipment may be of interest. We will therefore try to obtain the next-of-kin’s consent to perform medical autopsy on patients that are admitted to hospital in Norway, but do not survive to hospital discharge.

### Steps to be taken to control or mitigate risks

- The physicians are all experienced clinicians, familiar and competent in Seldinger technique. All the personnel must participate in a structured training program and be certified before they can perform the procedure.
- The equipment is well known and used in a range of indications and patient ages.
- The equipment is small gauge, which limit the risk of local complications.
- Training facilities is available at all study sites.
- All emergency services, first responder services and physician manned local out-of-hours emergency facilities will receive extensive information of the procedure.

## Summary of risk assessment

These interventions have always been associated with risk. Lower gauge of equipment provides reduced frequency of risk. All equipment will be handled by experienced personnel and always according to current CE/FDA regulations, hence the equipment is approved for use in the patient cohort.

We argue that this research study is justified, based on the rationale described in the background section and in this chapter.

# Adverse events

The investigator is responsible for the detection and documentation of events meeting the criteria and definition of an adverse event (AE) or serious adverse event (SAE).

Please note that in the present trial all included participants are expected to meet one or more criteria for serious adverse events. The nature of the patient population studied, the prehospital environment, severity of illness included, and multitude of medical interventions makes the AE reporting challenging. Several events that meet the AE criteria are expected in the natural course of critical illness and the treatment offered to these patients.

Each adverse event (AE) will be assessed for seriousness, causality, severity and expectedness.

| **Seriousness** | As defined in the protocol |
| --- | --- |
| **Causality** | Not related |
|  | Unlikely |
|  | Possible |
|  | Probable |
|  | Causal relationship |
| **Severity** | Mild |
|  | Moderate |
|  | Severe |
|  | Life-threatening |
|  | Fatal |
| **Expectedness** | Expected event in ACLS or REBOA intervention |
|  | Unexpected adverse event |

## Definitions of adverse events and device deficiencies

The definitions in this chapter is based on those from *ISO 14155:2011 Clinical investigation of medical devices for human subjects — Good clinical practice*.

**Adverse event (AE)**

Any untoward medical occurrence, unintended disease or injury, or untoward clinical signs (including abnormal laboratory findings) in subjects, users or other persons, whether or not related to the investigational medical device (Figure 7).

Note 1: This definition includes events related to the investigational medical device or the comparator.

Note 2: This definition includes events related to the procedures involved.

Note 3: For users or other persons, this definition is restricted to events related to investigational medical devices.

**Serious adverse event (SAE)**

An adverse event that

a) led to death,

b) led to serious deterioration in the health of the subject, that either resulted in

1) a life-threatening illness or injury, or

2) a permanent impairment of a body structure or a body function, or

3) in-patient or prolonged hospitalization, or

4) medical or surgical intervention to prevent life-threatening illness or injury or permanent impairment to a body structure or a body function,

c) led to foetal distress, foetal death or a congenital abnormality or birth defect

Note 1: Planned hospitalization for a pre-existing condition, without serious deterioration in health, is not considered a serious adverse event.

**Adverse device effect (ADE)**

An adverse event related to the use of an investigational medical device

Note 1: This definition includes adverse events resulting from insufficient or inadequate instructions for use, deployment, implantation, installation, or operation, or any malfunction of the investigational medical device.

Note 2: This definition includes any event resulting from use error or from intentional misuse of the investigational medical device.

**Serious adverse device effect (SADE)**

An adverse device effect that has resulted in any of the consequences characteristic of a serious adverse event

**Unanticipated serious adverse device effect (USADE)**

A serious adverse device effect which by its nature, incidence, severity or outcome has not been identified in the current version of the risk analysis report

Note 1: Anticipated serious adverse device effect (ASADE) is an effect which by its nature, incidence, severity or outcome has been identified in the risk analysis report.


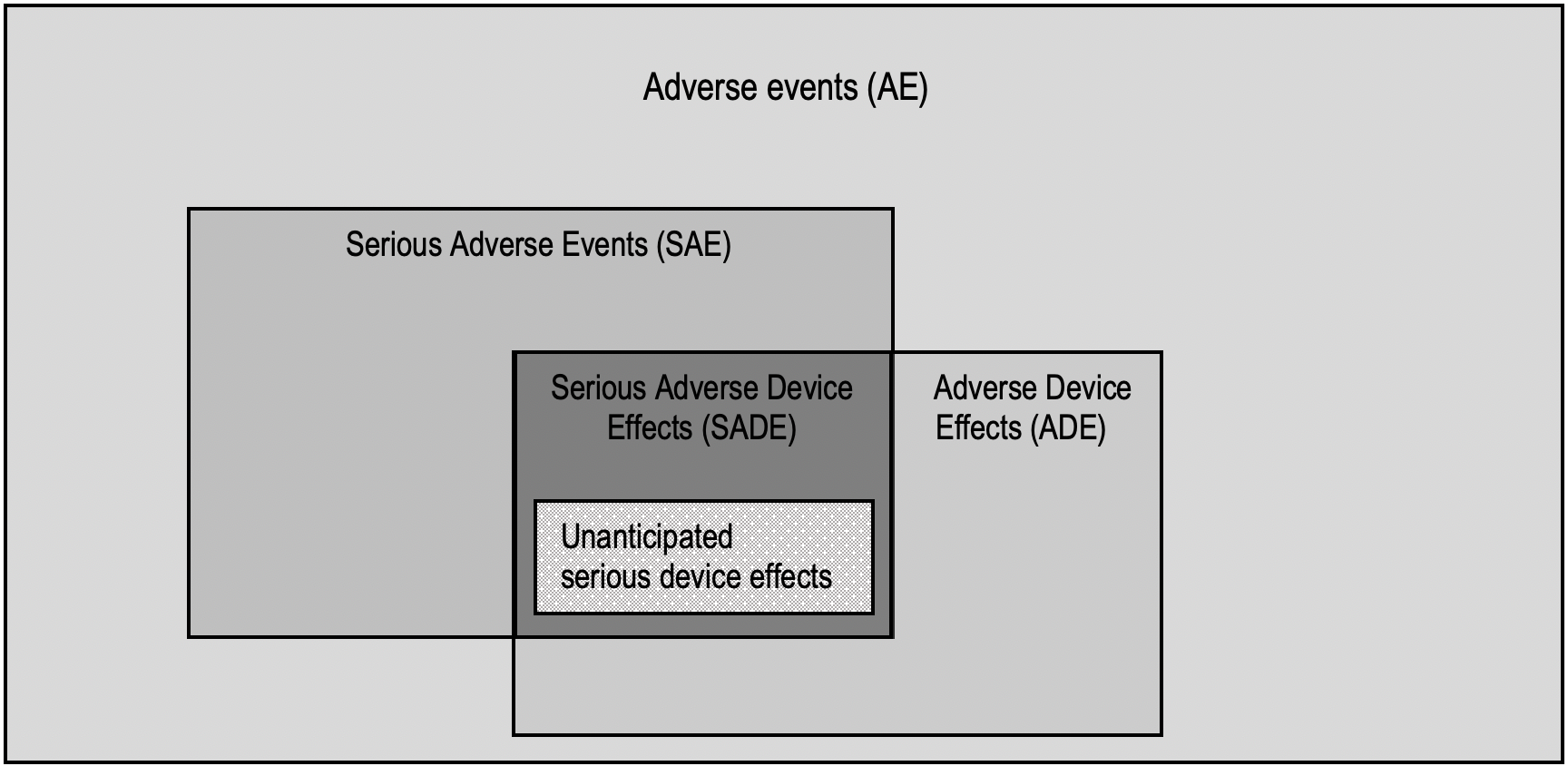


*Figure 7. Adverse events*

## Adverse events in the REBOARREST trial

Death is the most common outcome after cardiac arrest. Hence, we do not report death as a serious adverse event but register it as an expected outcome in the CRF.

All patients that achieve sustained ROSC will be transported to hospital and most will receive normal post-ROSC treatment, which include prolonged hospitalization and need of advanced medical and/or surgical interventions. It is therefore expected that nearly all subjects that achieve sustained ROSC will experience a serious adverse event after the definitions in this trial.

These SAEs are pre-specified outcomes and will not be reported but will be registered. We will therefore only report unexpected SAEs and SADEs/USADEs (events suspected related to the REBOA catheter and the handling of this equipment) to the sponsor and appropriate medicinal authority.

Adverse events will be documented in the CRF by the site investigator after the interview with the performing physician. If the patient is admitted to hospital, the SI will review the patient journal for documentation of adverse events 30 days after randomisation or if the patients dies prior to this. Adverse events that occur after this time will not be reported.

## Assessment of seriousness

The SI will assess the seriousness of the event according to the criteria defined in section 14.1. Please note that according to section 14.2, death and a number of serious complications are suspected, and will therefore not be reported as SAEs/SADEs.

## Assessment of causality

This section is based on MEDDEV 2.7/3 revision 3 from the European Commission for the purpose of this trial.

1. **Not related:** relationship to the device or procedures can be excluded when:

- the event is not a known side effect of any intervention in this trial
- the event has no temporal relationship with the intervention in this trial
- the serious event does not follow a known response pattern intervention in this trial
- and is biologically implausible;
- the serious event can be attributed to another cause (e.g. an underlying or concurrent illness/ clinical condition, an effect of another device, drug, treatment or other risk factors);
- harms to the subject are not clearly due to use error;

In order to establish the non-relatedness, not all the criteria listed above might be met at the same time, depending on the type of device/procedures and the serious event.

1. **Unlikely:** the relationship with the intervention in this trial seems not relevant and/or the event can be reasonably explained by another cause, but additional information may be obtained.
2. **Possible:** the relationship with the use of the investigational device or intervention is weak but cannot be ruled out completely. Alternative causes are also possible (e.g. an underlying or concurrent illness/ clinical condition or/and an effect of another device, drug or treatment). Cases were relatedness cannot be assessed or no information has been obtained should also be classified as possible.
3. **Probable:** the relationship with the use of the investigational device seems relevant and/or the event cannot reasonably be explained by another cause, but additional information may be obtained.
4. **Causal relationship:** the event is associated with the intervention in this trial beyond reasonable doubt when:

- the event is a known side effect the intervention in this trial
- the event has a temporal relationship with the intervention in this trial
- the event involves a body-site or organ that the intervention in this trial are applied to or have an effect on;
- the serious event follows a known response pattern to intervention in this trial (if the response pattern is previously known);
- other possible causes (e.g. an underlying or concurrent illness/ clinical condition or/and an effect of another device, drug or treatment) have been adequately ruled out;
- harm to the subject is due to error in use;

In order to establish the relatedness, not all the criteria listed above might be met at the same time, depending on the type of device/procedures and the serious event.

## Assessment of severity

All AEs and SAEs will be assessed for severity, according to the Division of AIDS (DAIDS) Table for Grading the Severity of Adult and Paediatric Adverse Events, version 2.1 (July 2017).

The following guidelines will be used to describe severity.

Mild (Grade 1): Events that are usually transient and may require only minimal or no treatment or therapeutic intervention and generally do not interfere with the subject’s usual activities of daily living.

Moderate (Grade 2): Events that are usually alleviated with additional specific therapeutic intervention. The event interferes with usual activities of daily living, causing discomfort but poses no significant or permanent risk of harm to the research subject.

Severe (Grade 3): Events interrupt usual activities of daily living, or significantly affects clinical status, or may require intensive therapeutic intervention. Severe events are usually incapacitating.

Severe (Grade 4): Events that are potentially life threatening.

## Assessment of expectedness

The SI should assess expectedness for each SAE/SADE/USADE regardless of the causal relationship to the trial device.

Please note that death and a number of serious complications (including prolonged hospitalization and need of advanced medical and/or surgical interventions) are expected outcome after cardiac arrest and will not be reported as a SAE/SADE.

The expected adverse events of the REBOA catheters in use in this trial are mentioned in the instructions for use, appendix 29.3. Other catheter types may be available for use during the study period. These will be treated as recommended in the producers “instruction for use”. Eventual supplementary expected adverse event according to the producer of these catheter(s) will be registered as appropriate.

**Expected adverse events common to both treatments**

- Death
- Multi-organ failure
- Extremity ischemia
- Abdominal and/or pelvic ischemia that may/may not require surgical intervention
- Stroke (embolic or related to hypoperfusion)
- Paralysis
- Paraplegia, permanent or temporary
- Renal complications requiring renal replacement therapy

**Expected adverse events related to use of REBOA**

Adverse events related to access

- Vessel dissection, perforation, rupture or injury
- Paraesthesia
- Infection, hematoma and/or pain at insertion site, with/without need for surgical intervention
- Haemorrhage at insertion site requiring treatment
- Pseudo-aneurysm
- Arteriovenous fistula
- Air embolism
- Arterial thrombosis, dissection, stenosis and/or embolism
- Need for arterial bypass
- Need for amputation

Other adverse events

- Contrast reactions
- Balloon rupture
- Occlusion at some locations may cause arrhythmia
- Aneurysm rupture

**Expected adverse events related to standard ACLS**

- Cardiac events and/or injury
- Thoracic aortic injury
- Lung injury/bronchopleural injury
- Oesophageal injury
- Respiratory failure
- Rib fractures
- Sternal fractures
- Aspiration pneumonia requiring antibiotic
- Injury to the gastric ventricle or liver

## Period for recording adverse events

For each patient the standard period for collecting and recording AE will be from randomization and until admission to hospital.

For each patient the standard period for collecting and recording SAE will be from allocation and until the 30 day follow up visit. At the 30 day visit an assessment will be made where we specifically ask for all SAEs prespecified in this protocol. We will also assess discharge notes for other events that fulfil the SAE definition and that can be assessed by the study team.

For patients still admitted to hospitals at day 30, an effort will be made to secure access to main discharge note for assessment for any SAE that has occurred between day 30 and discharge.

Any information that comes to investigators attention for any included patient prior to the end of trial will be assessed according to this protocol.

## Recording of adverse events

Patients in this trial are critically ill and are expected to need a prolonged hospital stay, often in intensive care unit and with several interventions. Both the standard care prior to arrival in hospital and during their stay have significant risks for adverse events. Expected serious adverse events will be recorded in the CRF, but not reported to the sponsor.

Adverse events occurring between allocation and the end of treatment phase by pre-hospital services will be fully recorded. Events occurring between end of treatment by pre-hospital services and day 30 will only be collected by checking hospital discharge note and paper CRF filled in by the SI after contact with the admitted hospital. The SI will assess the event for seriousness, causality, severity and expectedness before the event is registered in the CRF.

**Adverse event/device effect: Reportable events flowchart**


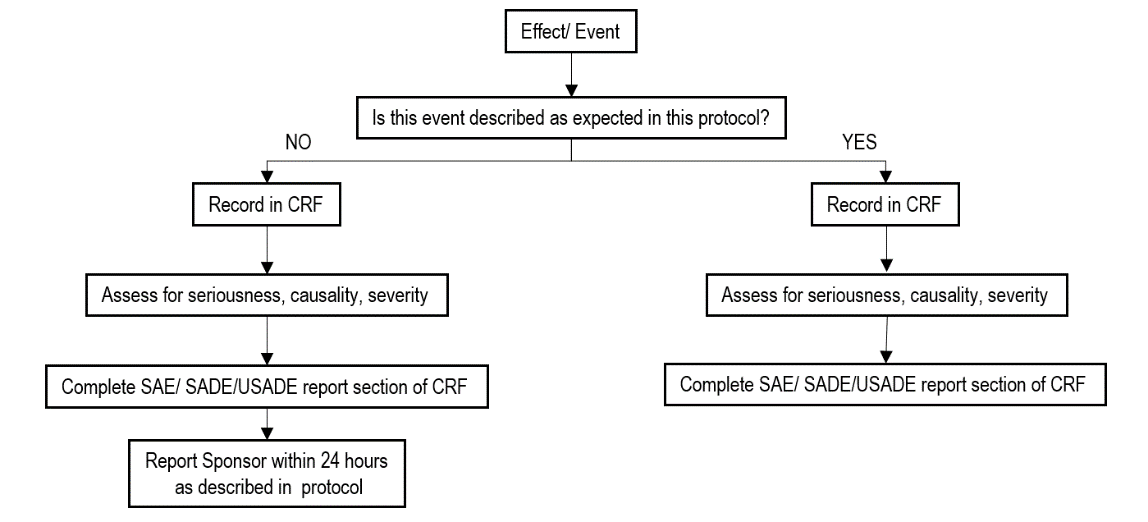


## Reporting of SAE/SADE/USADE

### Reporting from study site to chief investigator

A separate SAE/SADE/USADE form will be part of the CRF. Anyone suspecting a SAE/SADE/USADE should report this to the SI and/or the chief investigator as soon as possible and within 72 hours (3 days). The chief investigator makes the final assessment regarding seriousness, causality, severity and expectedness and need for further reporting.

A report of a suspected SAE/SADE/USADE must minimum include the unique study number of the participant, description of AE, initial assessment of AE for seriousness, causality, severity and expectedness and the identity of the person reporting.

### Reporting to appropriate medicinal authorities

Serious adverse device effects (SADE) and unanticipated serious adverse device effects (USADE) shall be reported to the Norwegian Medicines Agency by the responsible site. The incident report “Meldeskjema for virksomhet som bruker medisinsk utstyr” is found at the Norwegian Medicines Agency website (<https://legemiddelverket.no/medisinsk-utstyr/meld-korrigerende-tiltak-feil-og-uonsket-hendelse-med-medisinsk-utstyr>). SADE or USADE causing death or life-threatening illness or injury should be reported not later than 2 calendar days following the date of sponsor awareness, other SADE/USADE should be reported not later than 7 calendar days.

SADE and USADE should also be reported to the Norwegian Board of Health Supervision. As described on the boards webpage [www.helsetilsynet.no](http://www.helsetilsynet.no), this report should be emailed to [varsel@helsetilsynet.no](mailto:varsel@helsetilsynet.no).

This report should contain:

- The site investigators name and title
- Telephone number to the site investigator and information on when best to be contacted
- What special field in which the research is performed (emergency medicine) and which study site.

No health or identifiable information should be included in this report. The report should be sent as soon as possible after the SADE/USADE is discovered.

It is the responsibility of the chief investigator to ensure that each site investigator report such events from their site.

The current protocol describes the reporting procedures to Norwegian Authorities. Foreign study sites will have this section updated according to national procedures.

Each site must ensure compliance with the local hospital’s procedures relating the use of medical equipment.

## SAE/SADE/USADE advice

Contact phone numbers for SAE/SADE/USADE advice

Chief investigator (Dr Andreas Jørstad Krüger) +47 908 62 586

Project manager (Dr Jostein Rødseth Brede) +47 994 45 914

# Study management

This trial will be performed in accordance to the ICH GCP Notes for Guidance on Good Clinical Practice (CPMP/ICH/135/95). All study sites will have an own data monitoring system and data management and retention system. Individual patient data records will be handled according to the legislation of each participating country.

# Study Amendments

The research protocol may require amendments during the conduct of the study. Any amendment will be agreed upon between the sponsor and the project management. The amendments will be approved by REC and other relevant authorities at each site.

# Ethics Committee Approval

This trial will not start before ethics committee approval is granted. This approval applies for all Norwegian study sites.

Foreign study sites including patients must be approved by their regional or national ethics committee. The sponsor and project management will support the site investigator with the application to the ethical committee. It is the responsibility of the site investigator to ensure all appropriate approvals are in place prior to opening a site. All sites must comply with local regulations, national and EU law.

# Other Regulatory Approvals

Sites outside of Norway may be subject to national regulations in conjunction to European Union Regulation 2017/745 on medical devices. In such cases necessary approvals must be in place prior to opening a site. Application for such approvals is the responsibility of the site investigator.

# Trial insurance

In Norway, all patients who participate in the study are covered by Norwegian System of Patient Injury Compensation (Norsk Pasientskadeerstatning), a government agency subject to the Norwegian Ministry of Health and Care Services. See <http://www.npe.no/en/> for details.

Foreign study sites must ensure insurance cover for any included patients and provide the project management with documentation of this before the study site can enter the trial.

# Trial organisation

**Organisation**

The trial is sponsored by the Clinic of Cardiology, St. Olavs University Hospital. The main funder is the Norwegian Air Ambulance Foundation. The sponsor will communicate with the project management group or steering committee as appropriate. The data monitoring committee is independent from the project management and will communicate directly to the sponsor or the project management as appropriate (Chapter 22). Figure 8 describes this organisation.


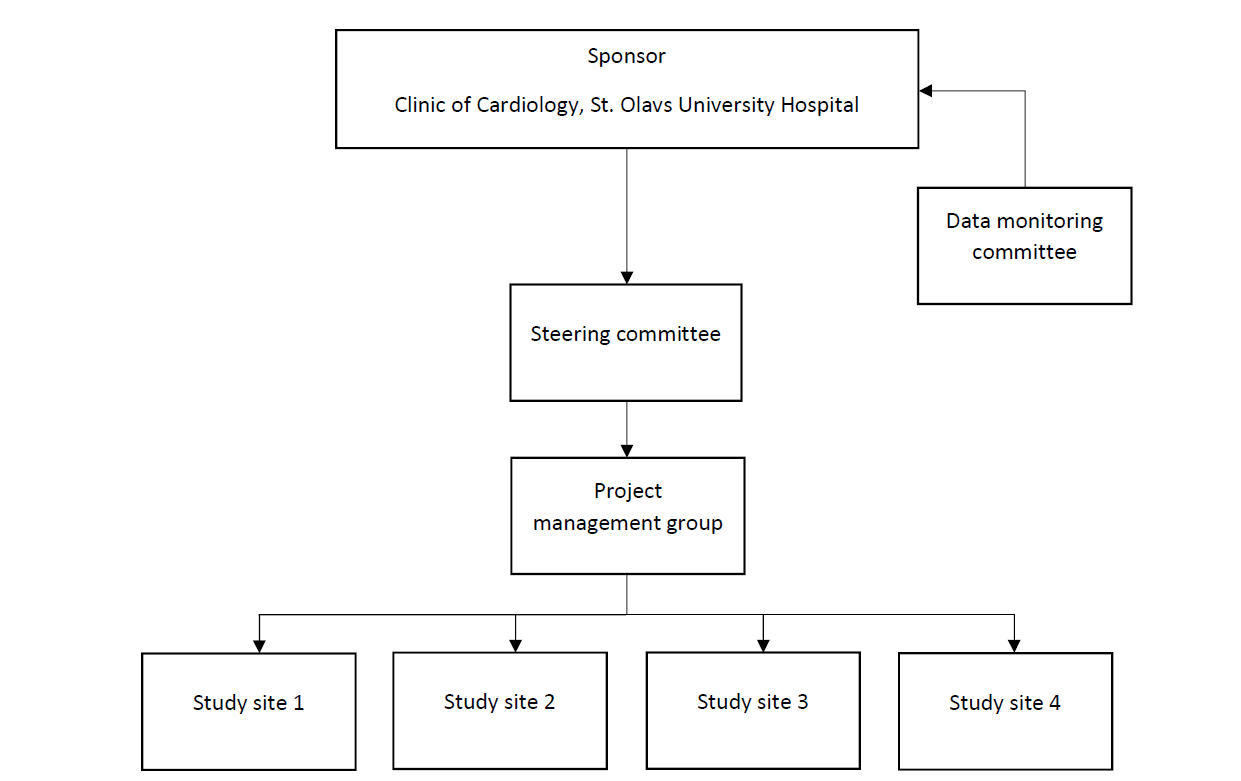


*Figure 8. Organisation chart of the REBOARREST trial.*

**The project management group**

This group consist of those involved in the day-to-day handling of the trial. They will have face to face meetings determined at need, but no less than yearly. Members of the project management are shown in chapter 1.1.

**The steering committee**

This committee consist of the project management, co-investigators (including research responsible site investigators) and members of project staff. The co-investigators are independent clinicians and experienced from running trials. The steering committee will oversee the trial. They will have face to face meetings determined at need, otherwise meet by video conferences or correspond via email.

**Administrative responsibilities**

Each study site has one research responsible person (site investigator, SI). This person may delegate tasks to another team member, a designee, as agreed with the project management. The SI is the administrative responsible person at the study site and have direct access to the steering committee for support. The SI manages education and sign-off at each study site, in cooperation with the steering committee. The SI is responsible for registration in CRF at each study site, that patient information is handled appropriately, and that necessary equipment is at hand.

The steering committee, in cooperation with the research responsible entities, will handle contact with the hospitals relevant for the study sites. This contact involves information to the relevant hospital departments and education of relevant personnel. The steering committee will ensure that all relevant parties at the study sites has signed an agreement that describe the responsibilities for each study site.

The research responsible institution’s responsibilities are described in chapter 21.

A third party, Clinical Research Unit (KlinForsk), Central Norway, will collaborate with the steering committee and the research responsible institution on several activities, that includes development of research protocol, randomisation, CRF, managements of adverse events, clinical study agreements and monitoring.

**Study site hospital**

As described in chapter 21.3, the relevant Head of Clinic (or other related employee) for each cooperating hospital that admit study participants will enter a written agreement describing each partners responsibilities and tasks before the study site and hospital can include any study participants.

## Statement that investigator is not allowed to deviate from the research protocol

The study will be performed in accordance with this research protocol.

## Procedures for recording, reporting and analysing protocol deviations

All research protocol deviations will be reviewed by the site investigator for impact on subject’s participation in the clinical study. The site investigator will notify the project management of deviations. All deviations will be reported to the appropriate regulatory bodies as required.

## Trial Master File

A paper-based trial master file will be maintained by the project management group and stored at the main study center, Rosten, Trondheim. Investigator site files will be made available to participating departments. These will be based on standards from NorCRIN. These will made available to monitor and authorities upon request.

# Study administration structure

## Research responsible

The sponsor is the research responsible institution. The responsibility includes

- To accommodate for proper organising, start up, conduct, communication, closure and post study management
- Enter into contract with suppliers and cooperating institutions
- Ensure routines for safe data handling and storage of research data
- Participant safety/safety reporting
- Ownership of data/open access/publish regardless of outcome

## Project management group

This committee is led by the project manager. The committee will

- Develop a research protocol and subject information sheet/consent form
- Ensure that study subjects are insured
- Handle and report study data and develop a data capture tool (case report form) for record of study data
- Ensure storage of study documentation, study file and institutional electronic document archive
- Ensure all communication with the regional ethics committee; application form, amendment notifications, ongoing report (if applicable) and end of study notification
- Ensure that cooperating institutions are trained on the study and procedures
- Register the study as applicable (ClinicalTrials.gov and kliniskestudier.helsenorge.no)

## Cooperating institutions and study sites

Each cooperating institution and study site will enter a written agreement describing each partner’s responsibilities and tasks. There will be a research site investigator (SI) responsible for the conduct of the study in each study site. This person acts on behalf of the project management group, as defined in the written agreement. This site investigator may delegate tasks to other team members, a designee, as agreed with the project management. This delegation should be in writing, see *Delegation Log* (appendix 29.9). The site investigator has the overarching responsibility to ensure that the team members have sufficient competence to conduct the delegated tasks. Only task can be delegated, not responsibilities. Each institution will be research responsible for the research conducted in own institution.

The responsible site investigator should

- Ensure internal acceptance and approvals for the study
- Facilitate internal agreements with cooperating units/internal partners
- Coordinate and ensure training of other team members in the institution
- Ensure that the study is conducted according to the approved research protocol
- Follow up the study subjects – obtain written informed consent, gather study data, handle adverse events/serious adverse events, including reporting these as appropriate, according to the internal routines at the institution and the study trial plan
- Ensure that ethical, medical, health related, scientific, personal data and information security issues/conditions are handled in the daily running of the study
- Ensure secure data handling and according to data management plan
- Report study status/progression to the project management
- Archive and store documentation (Investigator Site File)

# Data monitoring committee

An independent Data Monitoring Committee (DMC) will be established. The DMC will be governed by a charter based on the NorCRIN standard operating procedure.

The charter will describe the roles and responsibilities of the DMC, including the timing of meetings, methods of providing information to and from the DMC, frequency and format of meetings, statistical issues and relationships with other committees. The charter and DMC should be in place before the first patient is included.

# Monitoring

A monitoring plan specific to this study is held separately to the protocol. The monitoring plan outlines the level of monitoring to be performed for the trial and how this will be carried out. The level of monitoring is based on any perceived risks identified by the risk assessment. The risk assessment is done in collaboration between the sponsor and KlinForsk and will be based on the NorCRIN standard operating procedures.

# Funding

This trial is funded mainly by the Norwegian Air Ambulance Foundation. Other research grants or funders may be involved during the study period. None of the funders will contribute to the study design, collection, management, analysis or interpretation of data, nor writing of the report or decision to submit the report to publication. The sponsor retains full ownership of all data generated in the trial.

# Conflict of Interest

The sponsor has no conflict of interest in relation to any REBOA device or manufacturer in this trial. Any conflict of interest in relation to participating study sites must be accounted for in the cooperation agreements between the sponsor and that site.

JRB, AJK, AS, KT and MR are partly employed at or funded by the Norwegian Air Ambulance Foundation.

No other conflict of interest is reported.

# Data Sharing

**Metadata:**

The full study protocol, statistical analysis plan, information letter for consent and other trial documents will be published open access. The clinical study report and statistical analysis report will also be made openly available but may be altered to hide information that may lead to identification of individual study participants. These documents will be shared at HUNT Cloud. Full documentation can be made available for investigators in HUNT Cloud following conditions applicable to the REBOARREST data space as set forth by the project management to among others ensure the privacy of the study participants, compliance with participant consents and ethical approvals.

**Individual participant data:**

Individual level data will be made available to sponsor institutions on the REBOARREST data space in HUNT Cloud. Access will be controlled by a data processor agreement between the partner and funder.

Pseudonymous individual level data collected during the trial, will be made available on request to researchers from other scientific institutions by approval of the project management to ensure that usage is compliant with privacy and consent requirements as well as conditions for attribution and usage.

Data sharing with editors or peer-reviewers of scientific journals, conferences or the like will not require additional consent or data access agreement with sponsor, as such data will not be shared onward or used beyond reviewing this trial.

After data sharing, the sponsor must be acknowledged in any publication resulting from the shared data. For closer collaboration attribution based on the Vancouver Convention will apply.

# Publication policy

This protocol will be submitted for publication in an international peer reviewed journal.

On completion of the study, the results will be submitted for publication in an international peer reviewed journal. The results will also be submitted to the REC according to national regulations.

Authorship of the primary publication will be decided by the project management. Authorship is granted according to the Vancouver definitions^66^. All personnel who have contributed significantly but not fulfilled the criteria to authorship, will be mentioned under “Acknowledgements” in the manuscript. Study sites that recruit > 10 participants will be entitled to one name, > 20 participants will be entitled to one additional name, > 40 two additional names and > 60 three additional names in the author list. If a study site includes less than 10 patients, the site will not be entitled to an author name.

The main publication will report the primary and secondary endpoints. Detailed descriptions on hemodynamic properties will, because of the complexity, likely be submitted to a peer-reviewed journal as a separate manuscript. Authorship on these manuscripts will be granted according to the Vancouver definitions^66^.

# Bibliography

1. Brede Jostein Rødseth, Lafrenz Thomas, Klepstad Pål, et al. Feasibility of Pre‐Hospital Resuscitative Endovascular Balloon Occlusion of the Aorta in Non‐Traumatic Out‐of‐Hospital Cardiac Arrest. *J Am Heart Assoc*. 2019;8(22):e014394. doi:10.1161/JAHA.119.014394

2. Schulz KF, Altman DG, Moher D, CONSORT Group. CONSORT 2010 statement: updated guidelines for reporting parallel group randomised trials. *BMJ*. 2010;340:c332. doi:10.1136/bmj.c332

3. Chan A-W, Tetzlaff JM, Altman DG, et al. SPIRIT 2013 statement: defining standard protocol items for clinical trials. *Ann Intern Med*. 2013;158(3):200-207. doi:10.7326/0003-4819-158-3-201302050-00583

4. Tjelmeland IBM, Nilsen JE, Kramer-Johansen J, et al. Norsk hjertestansregister, Et register over gjenopplivningsforsøk i Norge. [Norwegian Cardiac Arrest Registry, a Registry of Resuscitation Attempts in Norway, yearly report 2017]. Published online 2018:71.

5. Engdahl J, Holmberg M, Karlson BW, Luepker R, Herlitz J. The epidemiology of out-of-hospital ‘sudden’ cardiac arrest. *Resuscitation*. 2002;52(3):235-245. doi:10.1016/S0300-9572(01)00464-6

6. Moriwaki Y, Tahara Y, Kosuge T, Suzuki N. Etiology of out-of-hospital cardiac arrest diagnosed via detailed examinations including perimortem computed tomography. *J Emerg Trauma Shock*. 2013;6(2):87-94. doi:10.4103/0974-2700.110752

7. Hasselqvist-Ax I, Riva G, Herlitz J, et al. Early cardiopulmonary resuscitation in out-of-hospital cardiac arrest. *N Engl J Med*. 2015;372(24):2307-2315. doi:10.1056/NEJMoa1405796

8. Dragancea I, Rundgren M, Englund E, Friberg H, Cronberg T. The influence of induced hypothermia and delayed prognostication on the mode of death after cardiac arrest. *Resuscitation*. 2013;84(3):337-342. doi:10.1016/j.resuscitation.2012.09.015

9. Norwegian Resuscitation Council. Norsk Resuscitasjonsråd. Norsk Resuscitasjonsråd. Accessed September 9, 2017. http://nrr.org/no/

10. Monsieurs KG, Nolan JP, Bossaert LL, et al. European Resuscitation Council Guidelines for Resuscitation 2015. *Resuscitation*. 2015;95:1-80. doi:10.1016/j.resuscitation.2015.07.038

11. Hutin A, Abu-Habsa M, Burns B, et al. Early ECPR for out-of-hospital cardiac arrest: Best practice in 2018. *Resuscitation*. 2018;130:44-48. doi:10.1016/j.resuscitation.2018.05.004

12. Manning JE, Murphy CA, Hertz CM, Perretta SG, Mueller RA, Norfleet EA. Selective aortic arch perfusion during cardiac arrest: A new resuscitation technique. *Ann Emerg Med*. 1992;21(9):1058-1065. doi:10.1016/S0196-0644(05)80645-6

13. Barton C, Manning JE, Batson N. Effect of selective aortic arch perfusion on median frequency and peak amplitude of ventricular fibrillation in a canine model. *Ann Emerg Med*. 1996;27(5):610-616.

14. Daley J, Morrison JJ, Sather J, Hile L. The role of resuscitative endovascular balloon occlusion of the aorta (REBOA) as an adjunct to ACLS in non-traumatic cardiac arrest. *Am J Emerg Med*. 2017;35(5):731-736. doi:10.1016/j.ajem.2017.01.010

15. Osborn LA, Brenner ML, Prater SJ, Moore LJ. Resuscitative endovascular balloon occlusion of the aorta: current evidence. *Open Access Emerg Med OAEM*. 2019;11:29-38. doi:10.2147/OAEM.S166087

16. Sesma J, Sara MJ, Espila JL, Arteche A, Saez MJ, Labandeira J. Effect of Intra-aortic occlusion balloon in external thoracic compressions during CPR in pigs. *Am J Emerg Med*. 2002;20(5):453-462. doi:10.1053/ajem.2002.32627

17. Nozari A, Rubertsson S, Wiklund L. Improved cerebral blood supply and oxygenation by aortic balloon occlusion combined with intra-aortic vasopressin administration during experimental cardiopulmonary resuscitation. *Acta Anaesthesiol Scand*. 2000;44(10):1209-1219.

18. Nozari A, Rubertsson S, Wiklund L. Intra-aortic administration of epinephrine above an aortic balloon occlusion during experimental CPR does not further improve cerebral blood flow and oxygenation. *Resuscitation*. 2000;44(2):119-127. doi:10.1016/S0300-9572(00)00132-5

19. Gedeborg R, Rubertsson S, Wiklund L. Improved haemodynamics and restoration of spontaneous circulation with constant aortic occlusion during experimental cardiopulmonary resuscitation. *Resuscitation*. 1999;40(3):171-180. doi:10.1016/S0300-9572(99)00021-0

20. Rubertsson S, Bircher NG, Alexander H. Effects of intra-aortic balloon occlusion on hemodynamics during, and survival after cardiopulmonary resuscitation in dogs. *Crit Care Med*. 25(6):1003-1009.

21. Paradis NA, Rose MI, Gawryl MS. Selective aortic perfusion and oxygenation: An effective adjunct to external chest compression-based cardiopulmonary resuscitation. *J Am Coll Cardiol*. 1994;23(2):497-504. doi:10.1016/0735-1097(94)90439-1

22. Paradis NA, Martin GB, Rivers EP, et al. Coronary Perfusion Pressure and the Return of Spontaneous Circulation in Human Cardiopulmonary Resuscitation. *JAMA*. 1990;263(8):1106-1113. doi:10.1001/jama.1990.03440080084029

23. Nozari A, Rubertsson S, Gedeborg R, Nordgren A, Wiklund L. Maximisation of cerebral blood flow during experimental cardiopulmonary resuscitation does not ameliorate post-resuscitation hypoperfusion. *Resuscitation*. 1999;40(1):27-35. doi:10.1016/S0300-9572(99)00003-9

24. Spence PA, Lust RM, Chitwood WR, Iida H, Sun YS, Austin EH. Transfemoral balloon aortic occlusion during open cardiopulmonary resuscitation improves myocardial and cerebral blood flow. *J Surg Res*. 1990;49(3):217-221. doi:10.1016/0022-4804(90)90122-I

25. Suzuki A, Taki K, Kamiya K, Miyake T. Cerebral blood flow during open-chest cardiac massage with occlusion of the descending aorta in dogs. *Resuscitation*. 1985;13(1):69-75.

26. Gedeborg R, C:son Silander H, Rubertsson S, Wiklund L. Cerebral ischaemia in experimental cardiopulmonary resuscitation — comparison of epinephrine and aortic occlusion. *Resuscitation*. 2001;50(3):319-329. doi:10.1016/S0300-9572(01)00350-1

27. Aslanger E, Golcuk E, Oflaz H, et al. Intraaortic balloon occlusion during refractory cardiac arrest. A case report. *Resuscitation*. 2009;80(2):281-283. doi:10.1016/j.resuscitation.2008.10.017

28. Deakin CD, Barron DJ. Haemodynamic effects of descending aortic occlusion during cardiopulmonary resuscitation. *Resuscitation*. 1996;33(1):49-52.

29. McGreevy D, Dogan E, Toivola A, et al. Endovascular resuscitation with aortic balloon occlusion in non-trauma cases: First use of ER-REBOA in Europe. *J Endovasc Resusc Trauma Manag*. 2017;1(1):42. doi:10.26676/jevtm.v1i1.18

30. Coniglio C, Gamberini L, Lupi C, et al. Resuscitative Endovascular Balloon Occlusion of the Aorta for Refractory Out-of-Hospital Non-Traumatic Cardiac Arrest - A Case Report. *Prehospital Disaster Med*. 2019;34(5):566-568. doi:10.1017/S1049023X19004795

31. Stub D, Bernard S, Pellegrino V, et al. Refractory cardiac arrest treated with mechanical CPR, hypothermia, ECMO and early reperfusion (the CHEER trial). *Resuscitation*. 2015;86:88-94. doi:10.1016/j.resuscitation.2014.09.010

32. Hirlekar G, Jonsson M, Karlsson T, Hollenberg J, Albertsson P, Herlitz J. Comorbidity and survival in out-of-hospital cardiac arrest. *Resuscitation*. 2018;133:118-123. doi:10.1016/j.resuscitation.2018.10.006

33. Brede JR, Kramer-Johansen J, Rehn M. A needs assessment of resuscitative endovascular balloon occlusion of the aorta (REBOA) in non-traumatic out-of-hospital cardiac arrest in Norway. *BMC Emerg Med*. 2020;20(1):28. doi:10.1186/s12873-020-00324-z

34. Nolan Jerry P., Berg Robert A., Andersen Lars W., et al. Cardiac Arrest and Cardiopulmonary Resuscitation Outcome Reports: Update of the Utstein Resuscitation Registry Template for In-Hospital Cardiac Arrest: A Consensus Report From a Task Force of the International Liaison Committee on Resuscitation (American Heart Association, European Resuscitation Council, Australian and New Zealand Council on Resuscitation, Heart and Stroke Foundation of Canada, InterAmerican Heart Foundation, Resuscitation Council of Southern Africa, Resuscitation Council of Asia). *Circulation*. 2019;140(18):e746-e757. doi:10.1161/CIR.0000000000000710

35. Dankiewicz J, Cronberg T, Lilja G, et al. Targeted hypothermia versus targeted Normothermia after out-of-hospital cardiac arrest (TTM2): A randomized clinical trial—Rationale and design. *Am Heart J*. 2019;217:23-31. doi:10.1016/j.ahj.2019.06.012

36. Broderick Joseph P., Adeoye Opeolu, Elm Jordan. Evolution of the Modified Rankin Scale and Its Use in Future Stroke Trials. *Stroke*. 2017;48(7):2007-2012. doi:10.1161/STROKEAHA.117.017866

37. Perkins Gavin D., Jacobs Ian G., Nadkarni Vinay M., et al. Cardiac Arrest and Cardiopulmonary Resuscitation Outcome Reports: Update of the Utstein Resuscitation Registry Templates for Out-of-Hospital Cardiac Arrest. *Circulation*. 2015;132(13):1286-1300. doi:10.1161/CIR.0000000000000144

38. Perkins GD, Ji C, Deakin CD, et al. A Randomized Trial of Epinephrine in Out-of-Hospital Cardiac Arrest. *N Engl J Med*. 2018;379(8):711-721. doi:10.1056/NEJMoa1806842

39. Haywood K, Whitehead L, Nadkarni VM, et al. COSCA (Core Outcome Set for Cardiac Arrest) in Adults: An Advisory Statement From the International Liaison Committee on Resuscitation. *Circulation*. 2018;137(22):e783-e801. doi:10.1161/CIR.0000000000000562

40. Fisher A, Andreasson A, Chrysos A, et al. An observational study of Donor Ex Vivo Lung Perfusion in UK lung transplantation: DEVELOP-UK. *Health Technol Assess Winch Engl*. 2016;20(85):1-276. doi:10.3310/hta20850

41. Chow S-C, Chang M. Adaptive design methods in clinical trials – a review. *Orphanet J Rare Dis*. 2008;3(1):11. doi:10.1186/1750-1172-3-11

42. Pallmann P, Bedding AW, Choodari-Oskooei B, et al. Adaptive designs in clinical trials: why use them, and how to run and report them. *BMC Med*. 2018;16(1):29. doi:10.1186/s12916-018-1017-7

43. Lorch U, Berelowitz K, Ozen C, Naseem A, Akuffo E, Taubel J. The practical application of adaptive study design in early phase clinical trials: a retrospective analysis of time savings. *Eur J Clin Pharmacol*. 2012;68(5):543-551. doi:10.1007/s00228-011-1176-3

44. Sample Size Calculator. Accessed May 8, 2020. https://clincalc.com/stats/samplesize.aspx

45. Schulz KF, Grimes DA. Multiplicity in randomised trials II: subgroup and interim analyses. *The Lancet*. 2005;365(9471):1657-1661. doi:10.1016/S0140-6736(05)66516-6

46. Pocock SJ. When (Not) to Stop a Clinical Trial for Benefit. *JAMA*. 2005;294(17):2228-2230. doi:10.1001/jama.294.17.2228

47. Bjelland TW, Dale O, Kaisen K, et al. Propofol and remifentanil versus midazolam and fentanyl for sedation during therapeutic hypothermia after cardiac arrest: a randomised trial. *Intensive Care Med*. 2012;38(6):959-967. doi:10.1007/s00134-012-2540-1

48. Brede JR, Lafrenz T, Krüger AJ, et al. Resuscitative endovascular balloon occlusion of the aorta (REBOA) in non-traumatic out-of-hospital cardiac arrest: evaluation of an educational programme. *BMJ Open*. 2019;9(5):e027980. doi:10.1136/bmjopen-2018-027980

49. Stannard A, Eliason JL, Rasmussen TE. Resuscitative endovascular balloon occlusion of the aorta (REBOA) as an adjunct for hemorrhagic shock. *J Trauma*. 2011;71(6):1869-1872. doi:10.1097/TA.0b013e31823fe90c

50. Dogan EM, Hörer TM, Edström M, et al. Resuscitative endovascular balloon occlusion of the aorta in zone I versus zone III in a porcine model of non-traumatic cardiac arrest and cardiopulmonary resuscitation: A randomized study. *Resuscitation*. 2020;0(0). doi:10.1016/j.resuscitation.2020.04.011

51. Krüger AJ, Skogvoll E, Castrén M, Kurola J, Lossius HM. Scandinavian pre-hospital physician-manned Emergency Medical Services—Same concept across borders? *Resuscitation*. 2010;81(4):427-433. doi:10.1016/j.resuscitation.2009.12.019

52. Sollid SJM, Rehn M. The role of the anaesthesiologist in air ambulance medicine. *Curr Opin Anaesthesiol*. 2017;30(4):513-517. doi:10.1097/ACO.0000000000000480

53. Hilty WM, Hudson PA, Levitt MA, Hall JB. Real-time ultrasound-guided femoral vein catheterization during cardiopulmonary resuscitation. *Ann Emerg Med*. 1997;29(3):331-336; discussion 337.

54. Getzen LC, Pollak EW. Short-term femoral vein catheterization. A safe alternative venous access? *Am J Surg*. 1979;138(6):875-878.

55. Sobolev M, Slovut DP, Lee Chang A, Shiloh AL, Eisen LA. Ultrasound-Guided Catheterization of the Femoral Artery: A Systematic Review and Meta-Analysis of Randomized Controlled Trials. *J Invasive Cardiol*. 2015;27(7):318-323.

56. Moore LJ, Brenner M, Kozar RA, et al. Implementation of resuscitative endovascular balloon occlusion of the aorta as an alternative to resuscitative thoracotomy for noncompressible truncal hemorrhage: *J Trauma Acute Care Surg*. 2015;79(4):523-532. doi:10.1097/TA.0000000000000809

57. Saito N, Matsumoto H, Yagi T, et al. Evaluation of the safety and feasibility of resuscitative endovascular balloon occlusion of the aorta. *J Trauma Acute Care Surg*. 2015;78(5):897-904. doi:10.1097/TA.0000000000000614

58. Teeter WA, Matsumoto J, Idoguchi K, et al. Smaller introducer sheaths for REBOA may be associated with fewer complications: *J Trauma Acute Care Surg*. 2016;81(6):1039-1045. doi:10.1097/TA.0000000000001143

59. Søvik E, Stokkeland P, Storm BS, Åsheim P, Bolås O. The use of aortic occlusion balloon catheter without fluoroscopy for life-threatening post-partum haemorrhage. *Acta Anaesthesiol Scand*. 2012;56(3):388-393. doi:10.1111/j.1399-6576.2011.02611.x

60. Taylor JR, Harvin JA, Martin C, Holcomb JB, Moore LJ. Vascular complications from resuscitative endovascular balloon occlusion of the aorta: Life over limb? *J Trauma Acute Care Surg*. 2017;83(1). doi:10.1097/TA.0000000000001514

61. Stensaeth KH, Sovik E, Haig INY, Skomedal E, Jorgensen A. Fluoroscopy-free Resuscitative Endovascular Balloon Occlusion of the Aorta (REBOA) for controlling life threatening postpartum hemorrhage. *PloS One*. 2017;12(3):e0174520. doi:10.1371/journal.pone.0174520

62. McComb BL, Munden RF, Duan F, Jain AA, Tuite C, Chiles C. Normative reference values of thoracic aortic diameter in American College of Radiology Imaging Network (ACRIN 6654) arm of the National Lung Screening Trial. *Clin Imaging*. 2016;40(5):936-943. doi:10.1016/j.clinimag.2016.04.013

63. Rogers IS, Massaro JM, Truong QA, et al. Distribution, Determinants,and Normal Reference Values of Thoracic and Abdominal Aortic Diameters by Computed Tomography (From the Framingham Heart Study). *Am J Cardiol*. 2013;111(10):1510-1516. doi:10.1016/j.amjcard.2013.01.306

64. Uchino H, Tamura N, Echigoya R, Ikegami T, Fukuoka T. “REBOA” – Is it Really Safe? A Case with Massive Intracranial Hemorrhage Possibly due to Endovascular Balloon Occlusion of the Aorta (REBOA). *Am J Case Rep*. 2016;17:810-813. doi:10.12659/AJCR.900267

65. Lov om obduksjon og avgjeving av lik til undervisning og forsking (obduksjonslova) - Lovdata. Accessed January 2, 2018. https://lovdata.no/dokument/NL/lov/2015-05-07-26

66. ICMJE | Recommendations | Defining the Role of Authors and Contributors. Accessed October 24, 2017. http://www.icmje.org/recommendations/browse/roles-and-responsibilities/defining-the-role-of-authors-and-contributors.html

# Appendices

## SPIRIT checklist

Attached document

## The modified Rankin scale

Score 0 No symptoms.

Score 1 No significant disability. Able to carry out all usual activities, despite some symptoms.

Score 2 Slight disability. Able to look after own affairs without assistance, but unable to carry out all previous activities.

Score 3 Moderate disability. Requires some help, but able to walk unassisted.

Score 4 Moderately severe disability. Unable to attend to own bodily needs without assistance, and unable to walk unassisted.

Score 5 Severe disability. Requires constant nursing care and attention, bedridden, incontinent.

Score 6 Dead

Good neurological status: score 0-3

Poor neurological status: score 4-6

## REBOA catheters product information and instruction for use

Attached documents: -ER-REBOA Catheter, Prytime Medical

-REBOA Balloon Catheter, Reboa Medical

## Label for REBOA procedure kit

Attached document

## On-scene checklist for pre-hospital physician

Attached document

## Interview template

Attached document

## Subject ID log

Attached document

## Screening log

Attached document

## Delegation log

Attached document
